# Stimulation-Stability and Preclinical Bench Testing of a New Auditory Nerve Interface

**DOI:** 10.21203/rs.3.rs-10887452/v1

**Published:** 2026-09-08

**Authors:** Taylor Stump, Stefan Strahl, Joseph Crew, Moritz Leber, Keno Hübner, Sandeep Negi, Justin Vankirk, Meredith E. Adams, Thomas Lenarz, Florian Solzbacher, Hubert Lim, Loren Rieth

**Affiliations:** 1 Department of Chemical and Biomedical Engineering, West Virginia University, Morgantown, 26506, WV, USA.; 2 MED-EL, GmbH, Innsbruck, Austria.; 3 Blackrock Neurotech, Salt Lake City, 84108, UT, USA.; 4 Department of Mechanical, Materials and Aerospace Engineering, West Virginia University, Morgantown, 26506, WV, USA.; 5 Department of Otolaryngology-Head and Neck Surgery, University of Michigan, Ann Arbor, 48109, MI, USA.; 6 Department of Otolaryngology-Head and Neck Surgery, University of Minnesota, Minneapolis, 55455, MN, USA.; 7 Department of Otolaryngology, Hannover Medical School, Hannover, Germany.; 8 Department of Electrical and Computer Engineering, University of Utah, Salt Lake City, 84112, UT, USA.; 9 Department of Biomedical Engineering, University of Minnesota, Minneapolis, 55455, MN, USA.

**Keywords:** Auditory, USA, implant, interface, neural, MEMS, lifetime, microelectrode, degradation

## Abstract

**Objective:**

Cochlear implants are the flagship sensory neural prosthetic, enabling improved hearing and speech perception in >1 million patients with hearling loss Zeng (2022). Despite over three decades of active work and innovations in cochlear implants, hearing performance for complex tasks such as speech-in-noise and music appreciation are still quite limited, putatively due to the challenge of selectively stimulating the auditory nerve with intracochlear electrodes through the bony cochlear wall. Our approach is to more selectively evoke hearing percepts through the development of an Auditory Nerve Implant (ANI) device that uses penetrating microelectrodes placed directly in the nerve for stimulation, and which will be evaluated through psychophysical testing as part of a first-in-human study. Stability and integrity of ANI electrodes during passive soaking and stimulation are needed to support initiation of the clinical trial, which we evaluated through thermal accelerated saline soak testing and stimulation stability testing.

**Methods:**

The ANI electrode stability in saline was assessed by accelerated aging at ≥ 67 °C with a 1.5-year equivalent endpoint using electrode impedance and microscopy to characterize outcomes. Stimulation stability testing evaluated electrode integrity after 6.3 billion pulses at 100 ***μ***A, which is the maximum clinical stimulation intensity, for a one-year equivalent use, and 1200 ***μ***A, for a high-dose protocol. Electrode impedance, longitudinal stimulation voltage, and microscopy outcomes were used to the quantify results.

**Results:**

All had stable impedances around 2 kΩ at the end of the accelerated soak test, except for three with higher impedances but still below the 70 kΩ failure criteria. All but one electrode survived stimulation stability testing. Optical and electron microscopy largely showed little to no change during testing, indicating no observable tip degradation, with the exception of one soak-test device.

**Conclusions:**

The outcomes from the studies indicate that ANI electrodes exhibit a high degree of integrity and impedance stability. Microscopy characterization proved effective for evaluating electrodes, providing complementary information to standard BSEM. This study suggests the ANI device tolerate a 1.5 year in-dwelling period for a 1 year perceptual study of stimulation evoked percepts, paving the way for clinical translation.

## 1 Introduction

Auditory neuroprosthetic devices, exemplified by cochlear implants (CIs), are used to treat severe to profound hearing loss and represent the most successful neural interface technology to date. Hearing loss is the most prevalent condition amongst sensory disorders, occurring in new-borns through geriatic patients resulting from multiple mechanisms Su and Chan (2017), Rh et al. (2020), Reed et al. (2023). Cochlear implants have recently surpassed 1 million patients treated as standard of care for severe or profound sensorineural hearing loss, and have been implanted in patients as young as 6-months Zeng (2022). CI placement in young prelingually deaf patients is highly beneficial, enabling the development of much higher levels of speech perception and language skills, while older patients benefit from restored social engagement to increase social fulfillment and decrease cognitive decline resulting from social isolation Lenarz et al. (2022).

The use of CIs to treat infants highlights the need for device lifetimes of multiple decades, as revision surgeries to replace devices increase risks for patients and have decreased hearing performance. Such device lifetimes have been achieved with macroelectrode-based devices such as the linear array of Pt-Ir electrodes patterned from laser-cut foils and potted in silicone used for CIs, and similar approaches used for deep brain, vagus nerve, and spinal cord stimulators. Achieving similar device lifetimes for microelectrode technologies has proven much more difficult as is evident by the current lack of marketed neuroprostethic devices using them, despite decades of effort Ahuja et al. (2011) Normann et al. (1999), and positive outcomes in a number of studies Hochberg et al. (2006) Musk (2019) Wairagkar et al. (2024).

While CIs are a remarkable success, there has been little improvement in key metrics such as speech-in-noise perception, music appreciation, and ability to localize sounds for more than 20 years Devocht et al. (2016),Fetterman and Domico (2002), McDermott (2004), Limb and Rubinstein (2012). One key limitation is the low electrical conductivity of bone < 100 mS/m Kipping et al. (2024), Zhang et al. (2025), Malherbe et al. (2015), Kalkman et al. (2014), Briaire and Frijns (2000). CIs use a linear array of electrodes placed within the scala tympani in the bony spiral canal of the cochlea to stimulate spiral ganglion neurons (SGNs) and evoke hearing percepts. The milieu surrounding the electrodes is perilymph, a relatively conductive physiological fluid. The combination of a conductive adjacent medium, need for current to pass through bony cochlear wall, and distance between electrodes and neurons decrease the specificity of neural activation and increases the stimulation threshold currents compared to electrodes in intimate contact with neurons Branner et al. (2001),Bierer et al. (2010). This results in the 12 to 24 electrodes used in contemporary CIs delivering 4 to 8 channels of independent percepts, which is less than the number needed for speech perception in challenging environments, refined pitch perception, and music appreciation Zeng (2004). Cochlear electrodes also cannot reach the apical turns of the cochlea where low frequencies (< 600 Hz) and their phase are perceived, limiting an important range of perception Greenwood (1961) Carlyon et al. (2025). Alternative approaches such as Auditory Brainstem and Midbrain Implants (ABIs and AMIs) are available as marketed devices or in development, but currently have more limited performance in speech perception tasks than CIs as recently summarized Lim and Lenarz (2015) Lim and Anderson (2006).

Our approach is to place a penetrating intra-neural electrode array directly in lateral aspect of the auditory nerve as it approaches the modiolus Lenarz et al. (2025). The Auditory Nerve Implant (ANI) device comprises the Blackrock Neurotech Auditory Nerve Utah Slant Array (AN-USA) and associated helical lead with Au wires, connected to an FDA-approved MEDEL SYNCHRONY2 cochlear stimulator. Additional details on the device design and fabrication will be presented in the methods. The AN-USA, shown in Figure 1, is a 3 × 5 shank variant of the Utah slant array (USA) with 12 of the 15 electrodes electrical connected, which in turn is a variation a of the Blackrock Neurotech NeuroPort Electrode, a cortical brain-computer interface with 510(*k*) FDA clearance. The AN-USA has shank lengths from 0.5 to 0.9 mm across its 1.2 × 2.0 *mm*^2^ area, using the slanted array geometry designed to provide localized stimulation across the cross-section of peripheral nerves Branner et al. (2001) Aoyagi et al. (2003). The specific AN-USA geometry is optimized for the human auditory nerve that is ~ 1.25 mm in diameter for normal hearing, but often closer to ~ 1.0 mm with hearing impairments, and has considerable variablility for both populations (Jaryszak et al. 2009),Herman and Angeli (2011),Kang et al. (2012). Stimulation with 10 × 10 Utah slant array (USA) in the above elbow aspect of median and radial nerves in volunteers has demonstrated recruitment of cutaneous percepts with small receptive fields and thresholds in the low 10s *μ*A George et al. (2020) Wendelken et al. (2017) as part of studies to restore sensation to arm amputees. Changes in impedance, stimulation thresholds, and evoked percepts have been reported for USA electrodes in both animal models and human subjectsGeorge et al. (2020), Straka et al. (2018), Wendelken et al. (2017) Recent and prior studies in pre-clinical feline models using the AN-USA and Utah array, respectively, have also demonstrated the ability to evoke percepts with thresholds in the low 10s *μ*A or less Thomas et al. (2026) Rousche and Normann (1998) Hillman et al. (2003).

The focus of this study is to determine the saline soak and stimulation stability of the ANUSA as part of the fully integrated ANI device, to collect data supporting its compatibility with a first-in-human study involving a 1.5 year in-dwelling period with 1 year of stimulation. Positive outcomes from thermal accelerated aging and stimulation stability testing from the study are necessary to support initiating a clinical investigation and meet program milestones. Optical and electron micrographs of all electrodes were captured before and after soaking or stimulation as part of physical characterization to monitor for degradation. The ANI devices were immersed in 1× PBS in sealed containers, connected by an inductive link, and initial electrode impedances collected with voltage matrix measurements. Devices for thermal accelerated aging (TAA) were maintained at 67 °C and 77 °C for the equivalent of 1.5 years, with periodic impedance measurement collection. The stimulation stability study was conducted at room temperature with 6.3 Gpulses (billion pulses), similar to our recent study Stump et al. (2025), using of 100 *μ*A pulses, representing the upper bound of known physiological safe stimulation levels with a charge delivery based on a 1-year clinical study. Additionally, a 12× higher current of 1200 *μ*A, a high intensity condition, was also evaluated using the same protocol representing the higher stimulation voltages that will occur in vivo and to explore the limits of stimulation stability. The electrode impedance and stimulation voltage were periodically measured during the stimulation protocol to monitor for changes associated with damage to the electrodes. Electrodes were imaged again at the study endpoint, tip metal quality was graded, and changes in the quality metric or surface morphology were analyzed. In addition, samples of the PBS solution for each test device were analyzed by inductively coupled plasma mass spectrometry (ICP-MS) to quantify ions in solution that result from stimulation induced degradation. This approach provides a thorough test of materials stability during passive soaking and with stimulation, to demonstrate the fully integrated ANI device will remain stable during our clinical trial.

## 2 Materials and methods

This study characterized two cohorts of fully integrated auditory nerve interface (ANI) devices as part of bench studies to evaluate 1) soak test stability and 2) stimulation stability of the electrodes. The thermal accelerated aging (TAA) study evaluated the soak test stability for devices (n=3) with a 1.5 year equivalent endpoint. The stimulation stability test evaluated if electrode degradation occurred while delivering the maximum quantity of charge that would occur during a 1 year implant at the 100 *μ*A limit for clinical investigation (n=6), and high-intensity conditions of 1200 *μ*A (n=5). All AN-USA devices were characterized before and after each respective test using optical microscopy and scanning electron microscopy (SEM). Table 1 summarizes the experimental protocols used for each device and distribution of electrodes used for them.

### 2.1 ANI Device Integration

ANI devices are fabricated by the integration of an AN-USA and the associated helically-wound gold wire lead from Blackrock Neurotech with a SYN-CHRONY2 cochlear stimulator from MEDEL. The AN-USA is a 3 × 5 shank variant (Figure 1a) of the 10×10 shank Utah slant array (USA), which in turn is based on the NeuroPort Electrode Utah Array (UA) from Blackrock Neurotech, a cortical brain-computer interface with 510(*k*) FDA clearance. The AN-USA has shank lengths from 0.5 to 0.9 mm and is approximately 1.2 × 2.0 *mm*^2^ across the array’s face where the 1.2 mm dimension is across the nerve and there is a 0.4 mm shank pitch. A new feature, the *Fin*, was added to improve surgical handling with forceps when placing the device in the auditory nerve under a surgical microscope and in the confined nerve exposure available.

The *Fin* was developed based on feedback and collaborations with the project surgeons through many cadaver surgeries used to develop the translabyrinthine approach we have adopted. The structure is implemented by attaching and overmolding a laser cut and structured 50-*μ*m-thick Pt-Ir (90-10) foil to the backplane of the array. It is 1 mm in width, and a 1 mm portion extends normal to the array backplane, while a 2 mm portion extends along the backplane of the array to provide adequate mechanical coupling and allow the device to tolerate the loadings and surgical handling used to place it. This design was then tested through a series of cadaver surgeries, determined to notably improve surgical handling and facilitate placement of the AN-USA in the nerve, and adopted as the design to be used for our clinical trial.

A second set of ANI device improvements involve the lead used to connect the AN-USA to the SYNCHRONY2 stimulator that can be seen in Figure 1b. The lead uses helically coiled and overmolded wires to connect the electrode array and stimulator, and is fabricated as part of the AN-USA assembly by Blackrock Neurotech. Development of the helical lead from the straight lead used for Neuroport devices was motivated by 1) the necessity to use Au-Pd (99-1) wires for wirebonds to the Utah array, 2) the need to meet mechanical test standards for the lead, 3) ability to control the lead’s mechanical characteristics, and 4) implementation of surgical handling structures.

The design and surgical handling characteristics for the lead were developed and validated across >20 human cadaver surgeries, large animal pre-clinical studies Thomas et al. (2024) Thomas et al. (2026), and have been used as part of intraoperative studies that will be the focus of a future report. The lead body is comprised of 12 insulated Au-Pd (99-1) 25 *μ*m wires, one for each connected electrode, and two insulated Pt-Ir (80-20) 76.2 *μ*m wires. The Pt-Ir wires are not electrically connected and are used to control the lead’s mechanical characteristics to achieve a balance between mechanical compliance and a degree of mechanical memory. The design enables shaping the lead to control forces on the array facilitating retainment in the nerve without causing mechanical trauma to it. The Au and Pt wires are helically wound together and overmolded in medical-grade silicone to form the lead. The AN-USA is at the distal end of the lead, and a custom developed interposer used to integrate with the MEDEL SYNCHRONY2 stimulator forms the proximal aspect. The lead is 1.3 mm in diameter, conforming to the existing CI lead, facilitating the use of existing stimulator overmolding hardware, materials, and processes.

The lead will be retained in a bone groove formed by the surgeon to decouple external mechanical forces from the array and to help position it for nerve insertion, motivating the need for a repeatable lead diameter. Near the distal end of the lead, a surgical handling *Wing* is formed as part of the silicone overmolding process, which can be observed in Figure 1b. The *Wing* is designed to allow torsional control of the lead, particularly using specialized forceps specifically designed to grasp the lead to control its position and rotation. The *Wing* design is based on structures used in cochlear electrodes from several manufacturers Roland (2005) Deep et al. (2019). An additional design element instantiated into the lead body is a 5 mm segment of straight lead located just proximal to the AN-USA. This segment has more mechanical memory, allowing it to be precisely bent and twisted to facilitate implantation and retainment of the electrode array in the auditory nerve. The length of this section and use of the two Pt-Ir wires were all carefully optimized over the course of >10 separate cadaver surgeries, for the electrode array handling characteristics when used with a translabyrinthine surgical access. Specific criteria for optimization of the mechanical characteristics for the distal lead included reliable handling and forming of the lead without directly handling the array or risking damage to the shanks, forming the lead to stably position electrodes for insertion, and achieving insertion and retainment when the electrode is placed in the nerve. To obtain reliable and stable positioning of the lead a precise 1.3 mm groove is formed in the mastoid using burr bits, and the lead is pressed into the groove. The position and orientation of the groove was also optimized and validated over the course of multiple cadaver surgeries, and was deployed during a clinical investigation involving placement of wired AN-USAs in nerves of human subjects under informed consent and being treated for acoustic neuromas.

An additional component of the new lead is the *interposer*, which enables integration of the AN-USA with the hermetic feedthrough pins of the SYNCHRONY2 stimulator Moritz Michael Leber et al. (????). The use of an interposer is needed to electrically and mechanically join the Au electrode array wires to the Pt hermetic feedthrough pins. The Au wirebonding process requires bonds at both the electrode array and terminal end of the wires. Our interposer approach was developed through multiple rounds of research, development, and validation, and utilizes a 100 *μ*m-thick alumina interposer with attached Pt pads and wires. MEDEL fabricates the interposer by first welding Pt foil pads to insulated and structured Pt wires and then using epoxy to attach these to the alumina interposer at precise spacings to match the feedthrough pins. Blackrock Neurotech then wirebonds the Au wires from AN-USA to the Pt pads on the interposer, and uses a thin layer of epoxy and silicone to strengthen the interposer. During integration of the ANI devices, the interposer is slotted between the feedthrough pins, and the remainder of fabrication process nominally follows standard MEDEL procedures. We adopt the MEDEL naming convention of ”K1” for Kanal 1 (channel), where the channel number is defined by the SYNCHRONY2 for the 12 connected electrodes. The remaining three electrodes are not connected, and designate as NC1 - NC3 (Figure 1a).

### 2.2 Optical and Electron Microscopy

Optical micrographs (OM) were collected with a VHX-7000 digital microscope (Keyence, Itasca, IL) including low magnification micrographs of the whole electrode array, and at 500× magnification for all individual electrodes both before and after the respective TAA and stimulation paradigms. ANI devices imaged using ring illumination, with the electrode shanks held at a roughly 45° pitch to decrease focus-stacking artifacts and improve contrast. Instrument controlled and processed Z-stacking was used to acquire extended depth of field micrographs for analysis. Micrographs collected before and after the stimulation paradigm were directly compared to determine if physical damage to the IrOx coating and electrode tip occurred. Tip metal damage was also quantitatively scored before and after testing, and comparisons made to determine the change in damage during testing. Optical imaging was also used to assign electrodes to categories of stimulated, control, or discarded, based on quality to utilize the most representative electrodes available for the studies. The Keyence analysis software and ImageJ (LOCI, University of Wisconsin) software were used for electrode surface area quantification. The length and radius of the exposed electrode were used in an expanded cone model to approximate the electrode surface areas Figure S1. The expanded model accounts for variations in the deinsulation position and blunt tips.

Backscattered electron microscopy was the primary modality used for analyzing the tip metal and electrodes due to the benefit of *Z*-contrast (atomic number) to discriminate between materials and quantify electrode quality. Images were taken using one of two scanning electron microscopes, a Zeiss Sigma VP (Zeiss, Dublin, CA) or a JEOL JSM-IT510 (JEOL, Peabody, MA). Some imaging was performed in high pressure (low vacuum) mode (1 × 10^−2^ mbar) to help mitigate sample charging artifacts without sample coating. A 20 kV primary beam energy was used with a working distance of 10 mm to facilitate capturing low magnification images. Overview images were taken before and after testing conditions at a 40× magnification for each array along with three progressively higher magnifications 500, 1250, and 2500× to capture overall electrode morphology and integrity, as well as more detail on the tip metal and electrodes. Supplemental materials Figure S5-S13 provides the 1250× micrographs used for analysis from all electrodes from before and after the respective testing paradigms.

Figure 2 presents representative OM and BSEM micrographs and identifies specific surface materials. These images highlight the color and contrast mechanisms associated with the IrOx electrode material, Parylene-C shank encapsulation, silicone backside and lead potting, and silicon for the shank and array backplane. In optical micrographs, the exposed IrOx electrode sites have a dark tone with some green iridescence, where evidence of topography, fractures, and damage can be appreciated. The Parylene-C is transparent and presents as a grey tone like the silicon below it, or a darker gray when covering IrOx, and maintains a glossy sheen. The silicon is predominantly exposed at chip tips, and often has a silvery-grey tone with a finish that depends upon the surface roughness and character. Silicone is used for potting and is highly transparent, mostly revealing the underlying materials.

Contrast mechanisms for BSEM are complementary, and the technique offers much higher magnification and depth of field, making it a strong complement to OM. The exposed IrOx and Pt thin-film electrode materials have highlight tone due to their high *Z*, offering strong contrast with Parylene-C, silicon, and silicone, making identification of damage or changes precise. Silicon presents as a lighter mid-tone, while Parylene-C and silicone are darker mid-tones, which in combination with their spatial distribution facilitates precise materials and damage characterization.

The quality of the IrOx tip metallization and electrode integrity was scored on a scale from 1 (best) to 4 (worst), to quantify and compare the as-received electrodes to outcomes from the study endpoints, similar to a previously used method Bjånes et al. (2025). The electrode scores were compiled into heat maps to visualize their distribution and aid in analysis of changes. A pooled bootstrapped two-tailed statistical analysis (MATLAB, Massachusetts, United States) was run on the imaging scores to determine if significant differences are present or resulted from testing.

### 2.3 EIS, CV, and VTs

The electrochemical properties of the electrode-electrolyte interface are pivotal characteristics for the stimulation performance of AN-USA electrodes. Precise measurements cannot be made from fully integrated ANI devices, as the stimulator electronics lack the capabilities of a research potentiostat; therefore, electrochemical measurements were collected from AN-USA *assemblies* (Figure S2-S3) solder integrated to a simple header-pin PCB. AN-USA assemblies are the last stage for electrode arrays prior to integration with the stimulator, making them the most representative option for electrochemical measurements. These measurements are used to compare with published Utah array characteristics and enable more thorough analysis of electrical measurements collected using the SYNCHRONY2 stimulator. Measurements include electrochemical impedance spectroscopy (EIS), cyclic voltammetry (CV), and voltage transients (VT). All measurements were collected in 1× (0.15 mM) phosphate-buffered saline at room temperature with a *p*H adjusted to 7.4 and inside a grounded Faraday cage. Cyclic voltammograms were used to calculate the cathodic charge storage capacity (CSC_c)_, and VT measurements were used to measure the charge injection capacity (CIC).

EIS and CV measurements were taken with a Reference 600+ potentiostat (Gamry, Warminster, PA) in a 3-electrode configuration with an ANI working electrode, saturated (4M KCl) Ag|AgCl reference (Pine Research, Durham, NC), and an 0.414 mm diameter uninsulated Pt wire counter electrode. EIS spectra were collected from 0.1 to 10^5^ Hz with 10 points per frequency decade at the open-circuit potential using a 20 mV RMS sine wave. Cyclic voltammograms utilized a 5 mV step size and 50 mV/s sweep rate for potentials inside the water window for IrOx of −0.6 to +0.8 V versus Ag|AgCl for three cycles, where the second cycle is presented and analyzed. The electrode CSC_c_ (mC/cm^2^) was calculated by integrating the area under the cathodic portion of the CV curve Cogan (2008) Negi et al. (2010b). Voltage transient measurements were performed using a STG 4008 neural stimulator (MultiChannel Systems (MCS) Reutlingen, Germany) using the same 3 electrode configuration. Constant current pulses at 200 pulses per second (pps) for 1000 pulses with a pulse duration of 200 *μ*s were applied in increments increasing by 10 *μ*A until the maximum cathodic interface potential (*E_mc_*) reached the −0.6 V water window. Additionally, VTs were collected at 100 and 1200 *μ*A to estimate the voltage transients that occur during the performed Stim-Stab protocols and quantify the *E_mc_*. Voltage transients were recorded using an NI-9221 ADC (National Instruments, Austin, TX) at 100 kS/s controlled by custom Matlab code similar to recent publications Stump et al. (2025) Sun et al. (2023). The charge injection capacity (mC/cm^2^) was calculated based on nominal stimulator current and phase duration resulting in an *E_mc_* of −0.6 V.

### 2.4 Voltage Matrix and Impedance Measurements

Measurement of electrochemical characteristics (e.g. impedance) for electrodes from fully integrated ANI devices required the use of the SYNCHRONY2 stimulator. *Voltage matrix measurements* (VMM) were used to collect electrode characteristics and were performed using Expert Impedance Field Telemetry (E-IFT) function in the MEDEL clinical software Maestro v9.0 (MEDEL, Innsbruck, Austria), or custom developed Python code with the research interface box (RIB2) (University of Innsbruck, Innsbruck, Austria) libraries. These measurements utilized a USB-connected and powered MAX Programming Interface Box (MAXbox) and a MAX Coil S (MEDEL, Innsbruck, Austria) to inductively power and control the magnetically attached ANI devices as depicted in Figure 3. Measurements were performed in 1× PBS in 300 mL clean research-grade polypropylene containers with sealed lids to prevent evaporation and contamination of the electrolyte.

Voltage matrix measurements involve the stimulation of each electrode and sequentially measuring the voltage at the end of the anodic phase from the stimulated electrode and all other non-stimulated electrodes, resulting in an array of measurements as shown in supplemental materials Figure S4. E-IFT measurements for thermal accelerated aging were collected with Maestro using 75.5 *μ*A cathodic-leading pulses with 24.17 *μ*s phase-widths and a 2.1 *μ*s interphase. The voltage was measured at the end of the anodic pulse and Ohm’s law applied to calculate impedance. Voltage matrix measurements for the Stim-Stab paradigm were performed with custom Python code using the RIB2 libraries. These measurements used a slightly longer 26.25 *μ*s pulse width available with RIB2. The VMM were collected at 75.5 *μ*A during the 100 *μ*A Stim-Stab paradigm to agree with measurements made with Maestro. A series of currents 75.5, 100, 300, 600, 900 and 1200 *μ*A were used for each measurement cycle as part of the 1200 *μ*A Stim-Stab paradigm to allow linearity and saturation of the stimulation voltage to be evaluated.

### 2.5 Thermal Accelerated Aging

Thermal accelerated aging (TAA) was used to evaluate the integrity of ANI devices as part of pre-clinical testing aligning with the AAMI CI86:2017 standard from §20.1 Association for the Advancement of Medical Instrumentation (AAMI) (2017), and used to support approval of our clinical investigation. ANI devices will be implanted for 1.5 years as part of our clinical study, which is used as the endpoint for our TAA test. A cohort of three (n=3) fully integrated ANI devices were submerged in 1× PBS in clean polypropylene containers for a temperature-time progression of 67 °C for 10 days (equivalent to 80 accelerated days) and 77 °C for 33 days (equivalent to 528 accelerated days) yielding a total equivalent lifetime of 1.5 years. The acceleration factor *f* was based on Arrhenius behavior using the temperature increase Δ*T* relative to body temperature (37°C) which for many polymeric materials is evaluated based on *f* = 2^(Δ*T*/10)^ (ASTM) Hukins et al. (2008) Nguyen et al. (2021). The samples were maintained at ±0.5 °C of the setpoints in a HeraTherm laboratory oven (ThermoFisher Scientific, Langenselbold, Germany). Device impedances were measured daily using the E-IFT function in Maestro 9.0 with a pulse current of 75.5 *μ*A and phase duration of 24.17 *μ*s. The primary failure criterion was an impedance >70 kΩ, which corresponds to the highest impedance that maintains the ability to deliver 100 *μ*A, the curent limit for the IrOx AN-USA electrodes used with our clinical study. The secondary failure criterion is evidence of electrode material degradation based on optical and electron microscopy. Observed loss or damage of tip metallization will categorize the electrode as failed by the soak test. Note that electrodes K1,K2,K4,K5,K8,K10 from Device 3 had uncharacteristic damage due to handling while preparing samples imaging at the 1-year-equivalent time point.

### 2.6 Stimulation Stability

Stimulation stability (Stim-Stab) testing was performed with fully integrated ANI devices. The devices were placed in polypropylene containers as previously specified. Custom Python code utilizing RIB2 libraries to control a MAXBox and MAX Coil-S was used to conduct the Stim-Stab paradigm. Maestro v9.0 was used for endpoint E-IFT impedance measurements (see Section 2.4). Two separate stimulation conditions including 100 and 1200 *μ*A pulse intensities were used that represent the stimulation current limit for our clinical investigation and more highly accelerated paradigm, respectively.

Both paradigms performed 6.3 × 10^9^ biphasic charge balanced cathodic leading pulses with 200 *μ*s/phase and a 2.1 *μ*s inter-phase gap, resulting in 20 nC/ph and 240 nC/ph, respectively, using 1600 pulses per second (pps), schematically represented in Figure 4. Using the average electrode surface area from Figure 5 (3.68 × 10^−5^ cm^2^), charge densities were calculated as 0.54 mC/cm^2^/phase at 100 *μ*A and 6.52 mC/cm^2^/phase at 1200 *μ*A. Voltage matrix measurements were collected at the beginning, endpoint, and after every two million pulses (20.83 minutes) resulting in 3154 measurement increments. The failure criteria was specified as an abrupt increase in stimulation voltage (electrode impedance) >50% for an electrical failure, or additional tip metal damage observed when comparing micrographs from before and after stimulation.

The estimated upper limit of charge delivered during the one year of our clinical study over which stimulation will occur is 252 C. This charge is delivered in 1.5 months in the 8× acceleration for this Stim-Stab paradigm. The acceleration factors are 5.33× acceleration for 1600 pps frequency and 1.5× acceleration for running the test 24 hours/day. The large area (0.59 cm^2^) Pt-Ir (80-20) electrode built in to the stimulator body was used as stimulation return in all cases. Microscopy inspection of the as-received integrated AN-USA electrodes found a broad range of initial qualities; therefore, from the 12 of 15 electrically connected electrodes we chose four (4) electrodes to receive stimulation, four (4) to serve as unstimulated controls, and four (4) were discarded.

A cohort of six devices (*n*=6) was tested with for 100 *μ*A case, yielding 48 primary electrodes (24 stimulated and 24 unstimulated controls). Note that impedance measurements were collected longitudinally from discarded electrodes, and all electrodes including the not connected ones were imaged. One device had a significant number of shanks damaged during handling and another two had minor damage. The 1200 *μ*A paradigm used five (*n* = 5) devices where four devices were previously used for the 100 *μ*A condition, and the same set of electrodes were stimulated, served as controls, or were discarded. The fifth device was previously used for the TAA study, and was needed due to handling damage that occurred to shanks for Device 5 during the 100 *μ*A test, and the need to return Devices 8 and 9 for other tests. The stimulation paradigm was otherwise identical to the 100 *μ*A, except for the higher stimulation current, resulting in the delivery of 3024 C of charge. All voltage matrix measurements for the 1200 *μ*A study were collected at 75.5, 100, 300, 600, and 1200 *μ*A with pulse durations of 26.25 *μ*s to monitor the range of stimulation voltages for these conditions. The presented longitudinal stimulation voltage plots use the 1200 *μ*A current to highlight the voltages required for this level of stimulation. Some electrodes were near the stimulator compliance limit for 1200 *μ*A; therefore, voltage matrix measurements using 75.5 *μ*A are also presented, and were used to determine if abrupt voltage increases occurred. After the stimulation paradigm, samples of the PBS electrolyte were analyzed using inductively-coupled plasma mass spectrometry (ICP-MS) using the procedures specified in the following section.

### 2.7 Inductively-coupled Plasma Mass Spectrometry

Inductively coupled plasma mass spectrometry (ICP-MS) was employed to quantify trace metal ion concentrations at the parts-per-billion (ppb) level in the PBS from the 100 *μ*A paradigm. Analysis was not performed on saline from the 1200 *μ*A condition as results from the 100 *μ*A case were inconclusive, and analysis presented in the discussion suggests it has low sensitivity due to higher saline volumes needed for this protocol. The specific isotopes measured include ^29^Si, ^47^Ti, ^191^Ir, ^195^Pt, and ^197^Au, which represent the stimulator housing, AN-USA and stimulator electrodes (Si, Ti, Pt and Ir), and the lead wires (Au). All measurements utilized an Agilent 7800 (Agilent Technologies, Santa Clara, CA, USA) instrument with Ni cones to avoid measurement artifacts from Pt cones. Table 2 lists ICP-MS all operating conditions. All calibration and measurement samples were maintained in clean polypropylene containers and 5 mL of each sample were stabilized with 0.3125 mL of 0.8 M trace metal clean nitric acid (SIGMA-ALDRICH, 225711-475ML) after the study endpoint to maintain ions in solution. The internal standard solution consisted of 100 *μ*L each of 1000 ppm Sc and 1000 ppm In (Inorganic Ventures, Lakewood, NJ, USA) diluted in 9.8 mL of 0.8 M nitric acid. Matrix matched blanks were prepared with 50 *μ*L of internal standard mix and 9.5 mL of 1× PBS with 0.8 M nitric acid. Reagent blanks were prepared with 50 *μ*L of the internal standard, 5 mL of 0.8 M nitric acid, and 4.95 mL (>18 MΩ) deionized water. Sample solutions were made with 2 mL from the Stim-Stab test, 1.98 mL 0.8 nitric acid, and 20 *μ*L internal standard mix. Matrix matched calibration curves were made with 100 *μ*L of 100 ppm standards of Ti, Ir, Pt, and Au (Accustandard, Connecticut, USA) and 9.5 mL 0.8 M nitric acid. The limit of detection (LoD) was defined as three times the standard deviation of the matrix blank (3*σ*), and the limit of quantification (LoQ) as 10 times the standard deviation of the procedural blank (10*σ*). Calibration concentrations across all four standards were 0, 1.3±0.07, 5.4±0.21, 9.7±0.28, 26.8±0.66, 50.1±0.71, 75.6±0.53, 98.9±0.93 *μ*g/L (ppb). Sample measurements were taken with He gas to mitigate polyatomic ion interference.

### 2.8 Statical Analysis and Rigor

Electrode micrograph quality scores and stimulation electrode selection (*n*=4 per device) were independently assessed and agreed upon by subject matter experts. AN-USA devices were imaged in optical and electron microscopy to best replicate viewing angles, and facilitate accurate comparisons. Electrodes that were damaged by handling procedures were excluded from comparative analyses. Statistical comparisons on optical and BSEM scores were made using a two tailed bootstrap analysis with Bonferroni and Sidak corrections in MATLAB. The two-tailed bootstrap statistical analysis was capped at a floor value of *p* = 0.0001 due to computational runtime restraints. Before and after impedances of stimulation and control electrode groups (n = 4) were assessed using independent-samples Welch’s t-tests to determine whether there was a significant change in average impedance, significance denoted using conventional notation. Homogeneity of variance of pooled before and after stimulation groups were evaluated with a one way ANOVA Brown-Forsythe median. Device 2 and 4 outlier impedances were excluded from all statistical comparisons.

ICP-MS sample procedure consisted of an initial calibration curve followed by randomized method blanks, reagent blanks and samples in between four sets of internal quality controls. Isotopes of each target element were chosen to avoid spectroscopic interferences.

## 3 Results

### 3.1 Preliminary Electrode Characterization

The geometrical surface area (GSA) of the opened electrode sites and the electrochemical properties for the connectorized ANI assembly electrodes constitute baseline characteristics to evaluate if they are comparable to prior devices, and analyze their electrochemical properties. Quantitative optical microscopy was used to characterize the overall character, tip shape, deninsulation area, and IrOx integrity for the integrated AN-USA electrodes. All arrays reported here conformed with general design and quality expectations from optical inspection, including no fractured shanks, particle contamination, bubbles, or coarse defects, consistent with the fabrication and quality assurance inspection procedures used to produce them. The GSA has a strong influence on electrochemical and stimulation characteristics; therefore, optical micrograph (OM) were used to quantify it with results plotted in Figure 5.

The conical and truncated conical models are used to estimate the GSA for whole or chipped tip electrodes, respectively. Some electrodes had damaged tip metal as-received, as can be observed in heat map data in Figure 8 and from optical and electron micrographs in supplementary materials S5-S13. Silicon has an areal impedance >100× higher than IrOx at 1 kHz for Utah array geometries Caldwell et al. (2018), indicating the quantified surface areas are upper limit values for the effective surface area. Surfaces are presented in Figure 5 for the reference electrodes (AN-USA assemblies) and all tested ANI devices, along with colors and hatching to represent in which studies the devices were used. All GSAs, except for six of the electrodes from Device 9, are below 10 × 10^−5^ cm^2^, with many in the 2 to 4 × 10^−5^ cm^2^ typically reported for Utah arrays (Negi et al. 2010b), (Caldwell et al. 2018),(Stump et al. 2025). Device 9 has six electrodes with larger surface areas, which occur on the longer electrodes, and this is clearly visible in collected micrographs (supplementary materials S13). A number of electrodes had GSAs larger than 4 × 10^−5^, which can result in lower impedance and greater stimulation stability. Utilizing larger exposed electrode surface areas for the ANI devices was a design choice to help ensure electrodes could achieve the desired <70 kΩ impedances across a 1-year study in human subjects.

Electrochemical characteristics of the AN-USA electrodes were characterized by impedance spectroscopy (EIS), cyclic voltammetry (CV), and and voltage transients (VTs) to provide data used in analysis of Stim-Stab testing results and outcomes, and to establish they are consistent with prior reports. The combined Bode plots of impedance magnitude |*Z*|(Ω) and phase angle *φ* (°) are presented as halo plots where the outline represents standard deviation in Figure 6a. The 1 kHz impedance was 4.86 ± 1.81 kΩ and was near the knee in the spectra that delineates capacitive interface at lower frequencies and the flat resistive electrolyte access resistance at higher frequencies. The average |*Z*| agrees well with measurements from ANI devices presented in Figures 7 and 10 for the after stimulation measurements. The average phase angle for these as-received electrodes was −36.7 ± 2.6°, which indicates a mixture of capacitive and resistive character, and are consistent with contemporary reports of the impedance characteristics for Utah electrodes. The electrolyte spreading impedance dominates for frequencies >10 kHz based on the flatter impedance curve with an average value near 2 kΩ and phase angle near 15°. A small negative slope is observed in the region dominated by the access resistance for frequencies > 10^3^ Hz, which is consistent with prior results for electrodes that have not been *activated* by stimulation Stump et al. (2025) Negi et al. (2010a). Electrode *activation* during stimulation has been observed to flatten the high frequency part of the impedance spectrum and shift it to lower values across most frequencies Otto et al. (2006) Stump et al. (2025).

The averaged cyclic voltammograms presented in Figure 6b do not show strong redox peaks, which is consistent with capacitive interfacial character and is in-turn consistent with prior data from Utah arrays with IrOx tip metallizations. The CV data was also used to calculate the cathodic charge storage capacity (CSC_c_ mC/cm^2^) by integrating the area within the loop for cathodic currents, which had an average of 25.83 ± 3.62 mC/cm^2^. Though less than state-of-the-art CSC_c_ values for Utah arrays Cogan et al. (2008) Cogan et al. (2016), it is comparable to those reported in past studies.

Voltage transient (VT) measurements were collected to determine if the character, quantified metrics, and calculated charge injection capacity (CIC) are consistent with prior reports. A halo plot for the average VT (*n*=8) is shown in Figure 6c. Note that data from two electrodes were not included due to much higher access voltage (V_a_) values making them not representative of typical electrode characteristics. The VTs of three current conditions are shown: 100 *μ*A clinical study limit (blue), 1200 *μ*A high intensity condition (red), and ~300 *μ*A where the maximum cathodic potential (*E_mc_*) reached −0.6 V vs Ag|AgCl for CIC measurements Cogan (2008), Negi et al. (2009), Stump et al. (2025). Similar to prior reports Negi et al. (2010a), the proportion of *E_mc_* and *V_a_* are roughly similar in magnitude for the CIC voltage transients. The access resistance calculated from the VT at the water window is 2.4 kΩ, which agrees with the high frequency component of the EIS spectra, which corroborates the data. The calculated CIC for the electrodes was 1.70 ± 0.25 mC/cm^2^. This CIC exceeds the maximum injected charge density per phase of 0.5 mC/cm^2^ (100 *μ*A for 200 *μ*s/ph with a GSA of 4 × 10^−5^ cm^2^) to be used for the clinical study, but CIC values decrease significantly in vivo compared to in vitro Chen et al. (2014), Nguyen et al. (2026). The *E_mc_* values measured for the 100 and 1200 *μ*A stimulation conditions had average values of −0.20 and −1.94 vs Ag|AgCl, respectively, with the latter value well exceeding the −0.6 V for the water window. The shaded halo regions represent the standard deviation, which was larger for the highest current stimulation conditions. Much of the variation is in the capacitive aspects of the curve, and resulted from offsets due to access resistance differences. These measurements support that the geometrical surface area and electrochemical characteristics for the AN-USA electrodes are consistent to prior reports, and that their stimulation characteristics will nominally align with those used for prior cortical and peripheral nerve studies.

### 3.2 Thermal Accelerated Aging

The primary objective for the development of the ANI device is to evaluate the evoked percepts and performance from intraneural stimulation through a first-in-human study Lenarz et al. (2025). Demonstrated device stability is a critical requirement for initiation of our clinical study, which has an in-dwelling period of 1.5 years, making this the endpoint of our accelerated aging soak test. The SYNCHRONY2 stimulator used is a marketed medical device, so has demonstrated stability; therefore, our test focuses on the AN-USA component. Devices 1 - 3 were used for the TAA testing.

Longitudinal impedance measurements, OM, and BSEM were used to quantify the electrochemical and physical stability of the electrodes. Devices were removed at 1 year equivalent for optical and BSEM inspection. The impedances collected using E-IFT measurements using Maestro are plotted longitudinally by days soaked in Figure 7. This figure also includes a second *y*-axis to present the percentage of surviving electrodes as a lifetime plot. The average impedance was 1.72±0.24 kΩ measured at 67 °C, which when measured at *room temperature* results in higher values of 13.29 ± 2.41, consistent with the higher resistivity of saline at lower temperatures. The room temperature measurements reasonably agree with the 7.94±2.88 kΩ saline measurements made prior to chronic large animal implants reported by Thomas et al. (2023).

A small and asymtoting downward trend in impedances can be observed during the first year (equivalent), which is consistent with electrode *activation* resulting from the use of 75.5 *μ*A pulses for impedance measurements. With the exception of one transient electrode Device 2 channel 7 at the beginning of the TAA test, all electrodes stayed well within specifications during year 1, which is indicated by the included lifetime plot.

The impedance for the remaining 0.5-years-equivalent are largely stable with the same values as the prior segment, with the exception of 4 electrodes. Device 2 channel 11 has a singular measurement at 62 kΩ for the first measurement after the 1-year SEM analysis, however, it returns to <5 kΩ stable levels. Channel 12 from that device also increases after SEM at the 382 to 432 equivalent days timepoint, but after 432 days, it trends downward to a steady value of 24 kΩ. Device 2 Channel 1 also increases markedly after SEM, but levels out at 30 kΩ, and is not quantified as failed. Device 3 channel 12 impedance spikes 88 kΩ after SEM, but then decreases to 37 kΩ for the remainder of the test. The impedance increases correlate with handling devices for imaging, but it is difficult to determine if the handling or imaging procedures caused the increases.

Optical microscopy was used for physical characterization of the ANI devices at the array and electrode level before, during, and after TAA, to evaluate tip metal quality and material degradation during soak testing. Figure 2 presents example images at the array and electrode level, and highlights the possibility to characterize electrode materials and their degradation. Micrographs of all electrodes collected before and after TAA are included as supplementary materials (Figure S5a, S5c, S6a, S6c, S7a, S7c) Heat maps quantifying the electrode and tip metal scores before and after soaking, and the changes that occur are presented in Figure 8, based on these micrographs. Images from the as-received electrodes show a notable degree of roughening for the IrOx tip metal based on surface morphology for the dark toned regions. Some tips have regions where the IrOx has delaminated leaving a smooth and silver-toned character. A statistical comparison across all nine as-received devices determined that the TAA cohort (Device 1 - 3) were more damaged than those used for Stim-Stab (OM *p*=0.0013, BSEM *p*=0.017). This damage can be more clearly appreciated in the BSEM data presented in the following paragraph. The metallization damage likely makes the TAA test more challenging due to uncontrolled exposure of the PtSi contact layer or silicon shank material, and is not representative for newer devices based on the statistical analysis, making this a worst-case study. Comparing the before and after accelerated soak test micrographs to identify changes to the electrodes was used to determine the stability and integrity of the electrode arrays. The heat maps indicate that Device 1 has no changes in tip damage, and Device 2 had two electrodes with an increase in damage (K11, and NC3). Careful comparison of the before and after micrographs supports this assessment. Device 3 has six electrodes (6/15) that experienced some tip metal degradation during the soak testing, when the heat maps and micrographs are compared, with electrodes 2, 4, 6, 9, 10, and 12 having worse metallization after soak testing. An increase in the shiny silver surface character, where the darker IrOx has delaminated is the most common failure mode. The BSEM analysis offers additional insights regarding initial tip metal quality that help in analysis of outcomes from the TAA testing. Note that the measured impedances for electrodes from Device 3 mostly did not change, except for electrode 12.

Backscattered scanning electron microscopy (BSEM) analysis provided significant additional insights regarding the initial electrode condition, and changes that occurred during our TAA paradigm. Heat maps depicting the electrode scores from before and after soak testing are presented in Figure 8, and are based on micrographs panels in supplemental materials S5b, S5d, S5f, S6b, S6d, S6f, S6h, S7b, S7d, S7f, For the open electrode area, the lightest tone is IrOx, midtone is the PtSi contact layer, and the darker tone is silicon revealed due to chipped tips, while the Parylene-C has the darkest tone. Device 1 has a number of electrodes with buckled IrOx that features fractures and prominences consistent with damaged adhesion. The mid-tones in regions around the buckled IrOx indicates the PtSi contact layer is retained. Additionally, electrode K3 has a chipped tip, based on exposure of the darker toned regions and truncated tip. Device 2 has more chipped tips, but a lower degree of buckling. Device 3 had a higher degree of buckling and delamination prior to soak testing.

A detailed comparison of the before and after BSEM images for Devices 1 and 2 reveals that no significant differences were observed after soak testing. The optical micrographs of Device 2 electrode 2 had revealed some apparent delamination of IrOx at the electrode tip, suggesting that change might have occurred between the characterizations. BSEM confirms that Device 3 fared worse during soak testing, agreeing with the 6/15 electrodes damaged. Backscattered contrast reveals that the primary failure mechanism is delamination of the buckled outer IrOx film, leaving the electrode surface with mid-tone contrast indicating the PtSi contact layer. Several electrodes such as K2 show darker mid-tone contrast on aspects of the electrode consistent with exposure of the underlying Si shank. This suggests that some degradation of the PtSi contact layer can occur in situations that yielded IrOx buckling, but mechanistic details would take more extensive and detailed characterization. The only electrode from Device 3 to experience a dramatic increase in impedance during soak testing was K12. This suggests that the PtSi contact layer provides reasonable impedance characteristics, and that microscopy inspection is a valuable quality analysis tool, with both optical and to a larger extent BSEM offering data that can characterize the state of the electrodes.

### 3.3 Stimulation Stability Testing

Stimulation of the auditory nerve to study evoked percepts are central to first-in-human study we are pursuing, which necessitates stimulation stability tests for the electrodes. While the device may be in-dwelling for up to 1.5 years, a stimulation period of 1 year was chosen to account for surgical recovery times and limited research session device use early in the study. Our estimated highest dose delivered in 1 year is 252 C, based on use of 16 hours/day, 300 pps, at 100 *μ*A pulses lasting 200 *μ*s/phase. The 100 *μ*A paradigm represented the maximum current we will utilize for the clinical study, while the 1200 *μ*A paradigm was a large amplitude test generating much higher stimulation voltages and delivered 3024 C. Longitudinal electrode stability was characterized by using VMMs, optical and electron microscopy, and trace analysis for degradation byproducts. Physical characterization is reported through 1) high-level heat maps of electrode quality Figure 8, 2) a set of representative OM and BSEM micrographs 9, and 3) a comprehensive set of OM and BSEM images of all electrodes in the supplementary materials S5-S13.

Starting with a comparison of beginning and endpoint impedances, values for stimulated and control (unstimulated) electrodes are presented in Figure 10 to evaluate overall trends during the 100 and 1200 *μ*A studies. During the 100 *μ*A stimulation of Devices 4 - 9 the median impedance for electrodes from Devices 5-8 had a statistically significant decreased (except Device 9 control electrodes) and often by >10 kΩ. Devices 4 and 9 impedances decreased much less for both stimulated and control electrodes. Note that impedance measurements for both stimulation and control electrodes utilized 75.5 *μ*A pulses, and can therefore contribute to electrode *activation* resulting in an impedance decrease. The impedances of all electrodes remained below 20 kΩ indicating no electrical failures occurred during this test. The pooled *Before* vs. *After* simulated and control groups (n=48) resulted in a significantly decreased standard deviation from 0.34 to 0.25 (*p* = 0.003).

For the 1200 *μ*A case, two notable differences include some electrodes with high impedances before stimulation, and the overall smaller decreases in impedance after stimulation. One stimulated electrode for Device 2 moved from 5 kΩ, a reasonable value, to >70 kΩ, consistent with failure of the channel. The control electrode (K1) was a failure at both the beginning and endpoints, consistent with its high impedance at the end of the TAA study. Device 4 has a stimulated electrode that starts high impedance, but comes down to a typical after stimulation value of 2 to 3 kΩ. Device 4 was also the only device with a larger impedance drop after stimulation with most electrodes decreasing by 5 to 10 kΩ. All the other devices had a <<10 kΩ decrease with stimulation. The pooled *Before* vs. *After* simulated and control groups (n=37) resulted in a more significantly deceased standard deviation of 0.26 to 0.058 (*p* ≤ 0.0001), compared to the 100 *μ*A case. Recall that Devices 4 - 7 were previously stimulated for the 100 *μ*A paradigm.

Drilling down, longitudinal data from the 100 *μ*A paradigm is presented in Figure 11 as three plots of voltage vs. number of pulses, including the initial 40 Mpulses and the full 6.3 Gpulses on 1.5 and 8 V scales. The 40 Mpulse detail is provided to highlight decreases in stimulation voltage that often occur early in the paradigm. Stimulated electrodes (green traces) often exhibit a very rapid decrease in voltage, quickly approaching an asymptote. Unstimulated *control* (blue traces) and *discarded* (brown traces) electrodes most frequently had a much slower voltage decrease, often more readily observed in the 6.3 Gpulse plots. All presented traces from viable electrodes, except Device 4 K10, decreased to values of 0.5 V or less at the study endpoint, consistent with the <5 kΩ impedances measured for the devices. The decrease of control and discarded electrodes is likely due to activation by the voltage matrix measurement pulses, which is consistent with their slower stimulation voltage decrease. Most importantly, no electrodes demonstrate the abrupt increase in voltage that has been previously observed with electrodes that fail during testing Stump et al. (2025). This suggests that all electrodes survived the test electrically.

Stimulation voltage vs. pulse plots for the 1200 *μ*A stimulation paradigm are presented in Figure 12, but use four columns to present voltages measured at (left) 75.5 and (right) 1200 *μ*A. Stimulation voltages measured at 1200 *μ*A for some electrodes from Devices 2, 4, and 5 are near the measurement limits making it harder to discern if abrupt increases in voltage occurred; therefore, companion measurements taken with 75.5 *μ*A are also provided. The reported voltages follow largely the same trends as the 100 *μ*A case, with a few notable exceptions. The stimulation voltages were clearly much higher in the right columns measured using 1200 *μ*A pulses, consistent with the much higher stimulation current. The early decrease in stimulation voltage is largely absent, likely due to most of the 1200 *μ*A samples having been previously used for the 100 *μ*A paradigm. Importantly, the Device 2 K5 electrode abruptly increases in voltage a little after 4 Gpulses indicating a failure; this result is also visible in Figure 9b. Further analysis of the VMM data for this electrode (not presented) reveals that no stimulation voltage (artifact) is measured by adjacent electrodes after the increase in stimulation voltage, which is most consistent with a lead wire failure. The other two electrodes that stayed at high voltages in the inset plots for 75.5 *μ*A plots across stimulation are K1 (control) and K12 (discarded), neither of which was damaged during the protocol.

The 75.5 *μ*A plots for devices 4 and 5 have some transient changes largely before the first 1 Gpulses, but these channels recover to more stable characteristics near the voltage levels at the beginning of the study for the remainder. These transients occurred for both devices being tested at that time, and are suspected to be transient artifacts. Additionally, Devices 2 and 4 each have two electrodes that remain at the measurement limit throughout the paradigm, all of which were either discarded or controls. The K1 electrode (stimulated) from Device 4 is initially very high impedance (voltage), but its voltage drops to very comparable values after roughly 0.5 Gpulses, and this electrode did experience damage as described in the following section. Device 5 electrode K12 (discarded) had high stimulation voltage throughout the 1200 *μ*A paradigm, and was a mechanically damaged electrode described below. All electrodes from Devices 6 and 7 were well behaved during this stimulation.

Physical characterization using OM and BSEM of all electrodes was performed before and after both the 100 and 1200 *μ*A paradigms, and our analysis most frequently uses the BSEM images to highlight changes due to more effective contrast mechanisms. The micrographs were used to evaluate electrode quality on a scale from 1 (best) to 4 (worst) based on the IrOx and structural integrity of the electrodes. The scores for all electrodes from before and after the stimulation paradigms and the changes in scores are summarized in Figure 8. Representative optical and electron micrographs are provided in Figure 9, which highlight some notable outcomes, while micrographs used to evaluate and score all electrodes are presented in the Supplementary Figure S5-S13. Starting with Devices 4-7, which were used with the 100 and then 1200 *μ*A paradigms, electrodes from Device 4 were significantly worse *p* ≤ 0.0001 by OM and BSEM in both its initial electrode scores and after stimulation (both paradigms) compared to other devices. The higher (worse) scores were primarily driven by chipped or fractured electrode tips, as can be observed in both OM and BSEM datasets Figure 8 , supplemental materials Figure S8. Electrode 5 was graded one point worse by OM after stimulation, but careful comparison of the BSEM images indicates no change was present after the 100 or 1200 *μ*A paradigms, consistent with the BSEM heat map for this electrode. Importantly, the BSEM score heat map indicates electrode K4 goes from a score 3 to 4 during the 1200 *μ*A paradigm. The BSEM image from after 1200 *μ*A (Figure S8f) for K4 (stimulated) shows a flake of IrOx tip metal has partially delaminated from the electrode, rounding of the exposed silicon features has occurred, and gap between the silicon and tip metal has formed. Electrode K1 (stimulated) also exhibited rounding of the exposed silicon surfaces and gap between silicon and the IrOx; however, no delamination was observed, and the changes were therefore subtle enough not to result in a score change.” This degradation mechanism is similar to the corrosion of silicon that has been observed from devices explanted after years of in-dwelling time in the cortex of human subjects Bjånes et al. (2025). The other stimulated electrodes for device 4 (K3 and K6) had no observed damage during the 1200 *μ*A stimulation, and none of the electrodes were damaged during the 100 *μ*A stimulation case, nor were the control or discarded electrodes in either case.

Device 5 experienced mechanical damage during handling to prepare the device for imaging after the 100 *μ*A paradigm resulting in chipped or fractured electrodes on 8/15 electrodes (K1, K2, K5, K7, K10, K12, NC1, NC2), and therefore does not provide valid data on electrode damage based on BSEM and OM analysis. Because this damage did not result from the stimulation paradigm, the electrode was scored as typical after stimulation, but the change in score was *excluded* to signify it resulted from non-representative means, a procedure we use consistently for all devices. After the 1200 *μ*A stimulation, the stimulated electrodes K9 and K10 experienced a small degree of degradation based on BSEM data, which cannot be seen in the OM data. Electrode K10 experienced rounding and erosion of the exposed silicon, but is not conclusive on if a separation opens between IrOx and silicon, similar to Device 4 electrodes. The damage is relatively modest given the significant tip chip that occurred prior to stimulation. Electrode K9 developed a small tip chip, while all other electrodes remained unchanged from imaging prior to stimulation.

For Device 6, two electrodes, K2 and NC1, were mechanically damaged during imaging preparation after the 100 *μ*A paradigm and therefore were *excluded* from the analysis of changes for both BSEM and OM. Careful comparisons of BSEM and OM from before and after both stimulation paradigms identified one electrode, K1 (stimulated), that changed during the 1200 *μ*A paradigm. The IrOx and electrode site for K1 was not delaminated or corroded, but a region of surface contamination with dark tone formed on the electrode. Recall that the stimulation voltages for all Device 6 electrodes were well within the expected ranges. Device 7 provides the simplest case where no changes were observed in BSEM or OM for either stimulation paradigm, and stimulation voltages stayed within typical ranges.

Device 2 was used with the 1200 *μ*A paradigm after TAA, where the only stimulation was from voltage matrix impedance measurements. The initial condition (after TAA) was slightly worse than other devices stimulated at 1200 *μ*A, except Device 4, with chipped tips and buckled metal defects being most prevalent. Only electrode K7 (discarded) was scored as worse by BSEM (no change in OM scores) after stimulation. Close comparison of before and after stimulation BSEM images for K7 show only subtle changes, and the worse score is potentially due to a more clear viewing angle of a small tip delamination. This electrode was one of the discarded channels as the poor initial state tip metal was unrepresentative. Recall that this device had one channel that failed during testing (K8: stimulated) and two that were open circuits for the entire 1200 *μ*A test (K1: control and K12 discarded), none of which correspond with electrode K7.

Two additional devices, Device 8 and 9, were used only with the 100 *μ*A stimulation paradigm. Each fully integrated device represents a significant investment; therefore, these devices were required for other testing and not used for the 1200 *μ*A paradigm. The BSEM data from before and after stimulation were collected with the Zeiss and JEOL SEMs, respectively, and the JEOL instrument provided a lower degree of *Z*-contrast than the Zeiss, resulting in the need for careful comparisons. Device 9 had no observed changes in electrodes by OM or BSEM, agreeing with the lack of voltage (impedance) increases in the longitudinal stimulation voltage (Figure 11). Device 8 had two electrodes (K1 (stimulated) and NC1 (no contact)) excluded from scoring for BSEM due to damage that resulted from handling the device, while the score for electrode K3 (control) worsened from a 1 to a 2. The damage to the electrode is minor, and might have become more easily appreciated by the difference in angle used to collect the image and changes in contrast mechanisms. Similar to Device 8 and all devices in the 100 *μ*A paradigm, there was no increase in voltage during the stimulation, and all voltages remained <0.5 V during the study.

Inductively-coupled plasma mass spectrometry (ICP-MS) was used to quantify trace ion concentration for elements used in the electrodes to evaluate electrode degradation. Measurements of Ti concentration were also collected due to its use in the stimulator housing. Although Si concentrations were measured to quantify the AN-USA substrate material, they did not produce stable calibration curves, and therefore the results were not included in the analysis. This is most likely due to the Si internal working quartz components. The results of the ICP-MS measurement after the stimulation condition 100 *μ*A are presented in Table 3. The limit of detection (LoD) and limit of quantification (LoQ) for the PBS matrix were carefully quantified and presented in the table. The measurements of ^191^Ir and ^197^Au were below the LoD, while some measurements of ^47^Ti and all for ^195^Pt gave measurable concentrations. The measured ^195^Pt concentration of between 0.54 - 2.26 ppb which was above the 0.120 ppb LoQ. The estimate change in Ir concentration upon dissolution of all exposed IrOx tip metallization for one electrode site would be ≈ 0.31 ppb, calculated from film thickness and the exposed geometric average surfacce area of 3.68 × 10^−5^ , using the full density of rutile IrO_2_ (*ρ* = 11.66 g/cm^3^) despite the porosity of the IrOx tip metal previous reported Negi et al. (2009) Sun et al. (2023). Dissolution of the IrOx from the four stimulated electrodes could result in an Ir concentration of 1.24 ppb, which does exceed the LoQ level in this extreme case, but is far less sensitive than the BSEM characterization. A similar situation is present for the PtSi contact layer, which is significant thinner than the IrOx layer, and Pt accounts for 50 at% of the material, resulting poor sensitivity, particularly relative to electron microscopy analysis. Device 4 and 6 had a measurable ^47^Ti concentration of 4.5 and 4.2 ppb respectively, while Device 5 had a measured concentration of 0.84 that was be low the LoQ of 1.5 ppb. The known stability of Ti implant enclosures, and the fact that only two devices exceed the LoQ by a small degree suggest that no unanticipated corrosion occurs.

## 4 Discussion

The core objective of device testing in this report is to thoroughly evaluate the electrode array stability for the ANI device as part of determining its suitability for a clinical investigation with up to 1.5 years in-dwelling and 1 year of stimulation. This testing aligns most closely with §20.1 ”Maintenance of integrity of materials for life of the implantable device” from the CI86:2017 standard Association for the Advancement of Medical Instrumentation (AAMI) (2017), which provides a high-level framework regarding avoidance of unacceptable hazards from materials degradation. Our study has three primary components that include 1) establish the baseline characteristics of the AN electrode array, 2) evaluate integrity of electrodes for up to 1.5 years in saline, and 3) quantify the stimulation stability of electrodes at both representative and very high stimulation levels.

Evaluation of the baseline device characteristics was an important first step for the study. The quantified electrode geometrical surface area (GSA) reported in Figure 5 agreed with prior reports for the Utah array, justifying that the results of this study are representative, as differences in surface area strongly affect outcomes from Stim-Stab testing. The design for ANI electrodes allows for small increase in tip GSAs to facilitate maintaining impedances <70 kΩ during the clinical study. Additionally, the GSA is important for interpretation of the baseline electrochemical data, where the impedance, CSC_c_, and CIC depend on the surface area. The impedance spectra, cyclic voltammograms, voltage transients, and the derived CSC_c_ and CIC for the connectorized AN-USA assemblies agree with published data Caldwell et al. (2018) Negi et al. (2010a) Nguyen et al. (2026). From the voltage transients, it is important to note that we appear to be stimulating with voltages well above the water window for the 1200 *μ*A condition, and delivering a considerable dose of charge under these conditions. A significant degree of electrode *activation* was observe from the Stim-Stab longitudinal voltages, whereas the electrochemical characterization only briefly stimulated electrodes; therefore it is possible that the *E_mc_* decreased during the stimulation paradigm to levels within the water window. This would require a marked drop in *E_mc_* from 1.94 to 0.6, so is considered unlikely, but cannot be directly measured with the SYNCHRONY2. The voltage transients were collected using a phase duration of 200 *μ*s, which was also used for the Stim-Stab pulsing. A 200 *μ*s phase duration is very long for modern cochlear implants, which typically use ~25 *μ*s pulses for high performance coding strategies (Lenarz et al. 2025), (Zappia et al. 1990), (Winn et al. 2012). Shorter pulses result in a lower voltage for a given current for electrodes like the AN-USA that have a degree of capacitive behavior at the electrode-electrolyte interface.

The second goal was to evaluate the the materials integrity and stability of electrode arrays and ANI devices using a thermal accelerated aging (TAA) study with an endpoint of 1.5 years in saline. All electrodes were classified as survived at the endpoint of the study based on their measured impedances, which served as the primary metric, marking an important milestone for our device. During the study four (4/36) electrodes which transiently crossed the 70 kΩ failure criteria and then decreased below the failure limit albeit at still elevated impedances near 30 kΩ. The passing impedances were observed despite Devices 1-3 having statistically higher (worse) electrode scores relative to Devices 4-9 used for the stimulation conditions indicating worse electrode and tip metallization character, largely due to *buckled* metallization damage and chipped tips. The most common trends in impedance for NeuroPort Electrodes implanted in the cortex of human subjects is for a near-term (~1 month) increase in impedances, followed by a longer-term trend to lower impedances attributed to the tissue response and encapsulation materials changes Downey et al. (2018) Bjånes et al. (2025)). The recent large animal pre-clinical study with a percutaneous version of the AN-USA found the in vivo impedance were relatively stable within the 13 to 41-week endpoints, but did have higher impedances with averages ranging from 25 to 68 kΩ and more variation compared to saline measurements Thomas et al. (2026)). The combination TAA and pre-clinical outcomes suggest there is are reasonable chances stable and useful impedances will be maintained for a clinical study with a 1.5 year in-dwelling period.

The electrode scores from OM and BSEM for the fully integrated devices are also important for interpretation of the outcomes from this study. The microscopy analysis comparing electrodes before and after soak did find that two and six electrodes from Device 2 and 3, respectively, were damaged during TAA resulting in worse scores, attributed to the clear vulnerability the *buckled* IrOx to delamination, the operative failure mechanism. The ability of electrodes to maintain passing impedances despite the observed damage suggests a degree of resilience, but impedances in vivo are notably higher than in saline Sun et al. (2023),Nguyen et al. (2026) and the mechanical forces the would experience are notably worse. This suggests that avoiding in vivo use of devices with buckled metal is advisable. A small change in the device integration process has eliminated the occurrence of buckled metallization making this unlikely for future devices, therefore these studies represent a worst-case scenario for device stability testing. Additionally, comparable electrode scores and ability to appreciate damage to the electrodes between optical and electron microscopy was another important finding, indicating OM is a viable tool for quality assurance inspections, which has been adopted for this project.

Pairwise analysis between the electrode device scores shows a statistically significant difference between Device 4 and all other devices as received in each stage of the study (Before, After TAA, After 100 *μ*A, and After 1200 *μ*A). All devices used for the TAA protocol (Devices 1-3) were statistically scored worse worse than those used for Stim-Stab (excluding device 4). While there are differences between tip scores of devices, there was only small changes that resulted from the soak and stimulation tests themselves, when electrodes damaged by handling are excluded.

The third component was to quantify the stimulation stability of the AN-USA electrodes with the goal of demonstrating their stability at the charge delivery levels expected for 1 year of stimulation in a clinical study. The 100 *μ*A stimulation condition represents the current limit that will be used in the clinical investigation, justified by seminal safety studies from the Bensmaia lab and multiple cortical and peripheral implants studies Rajan et al. (2015) Fernández et al. (2021) Koivuniemi and Otto (2011) Armenta Salas et al. (2018) Fifer et al. (2022) Flesher et al. (2016) Patrick-Krueger et al. (2024). Devices 4-9 were utilized for Stim-Stab with the primary metric being the absence of abrupt increases in stimulation voltages that result from damage to the IrOx. All electrodes in the stimulated category had consistent or decreasing stimulation voltages and passed the test electrically. Devices 4 and 5 had two and one electrodes, respectively, all of which were *discarded* and also had high voltages consistent with open circuits throughout the study, and do not provide any evidence of stimulation induced damage. When quantified as impedances, we noted that all values dropped, often by more than 10 kΩ, and most devices had impedances are 2 or 3 kΩ at the endpoint. Note that measurements for this study used 75.5 *μ*A pulses with 26.25 *μ*s phase durations resulting in a pulse duration of 55.26 *μ*s with the 2.1 μs interphase is included, roughly corresponding to a characteristic frequency of 18 kHz. This frequency is much higher than the typical 1 kHz used for impedance measurements, and at a much higher current both of which can contribute to small decreases in impedance measurements for this situation.

The decrease in impedance is consistent with the process of *activation*, a poorly understood process whereby electrochemical reactions decrease impedance Stump et al. (2025) Otto et al. (2006). The longitudinal voltages with time for Devices 7 and 8 (Figure 11 for the 6.3 Gpulse plot) highlight the differences in activation rates between stimulated and unstimulated electrodes (control and discarded). The green traces (stimulated) quickly decrease to values near 0.1 to 0.2 volts, whereas a more graduate decrease occurs for the blue (control) and brown (discarded) traces. This is consistent with the vastly lower charge delivery that occurs with only measurement pulses collected every 2 Mpulses for the simulated electrode. Nominally, the constant current pulses provided by the stimulator should generate consistent evoked activity, unless the activation process brings stimulation pulses initially beyond the compliance limit into compliance.

Microscopy characterization comparing electrodes after 100 *μ*A stimulation to before found that electrode materials and morphology were unchanged, except for electrodes damaged just before endpoint microscopy during preparation and handling. This was true even for Device 4 which had statistically significantly worse electrode tip metal scores than others in the cohort, due mostly to many chipped tips. These results suggest that chipped tips have some degree of resilience to stimulation.

The ICP-MS characterization unfortunately provided little insight as Ir concentrations in solution were below the LoQ. Estimating the maximum possible Ir concentration in solution with full dissolution of the tip metallization suggests well less than a 2 ppb concentration would result. The Pt values were also somewhat variable, and full dissolution of the tip metal would result in even smaller concentrations due to the thinner layer and that it deposited as a PtSi material. Additionally, ICP-MS measures ions in solution, and there is evidence that delamination is the process that results in IrOx loss. Ir and IrOx are extremely resistant to dissolution even in acidic solutions; therefore, it is unlikely delaminated IrOx would contribute to the measured concentration. Previous ICP-MS results with the highest charge delivered condition of 0.30 C for stimulation of IrOx electrodes from a Utah array found 0.22 ppb concentrations of Ir(Negi et al. 2010b). The much smaller saline volume used for stimulation, differences in IrOx character and processing, and differences in the stimulation paradigm, making comparisons difficult. The larger volume of PBS used to accommodate the full implant, and stimulation of only selected electrodes results in BSEM being a much more sensitive and precise method to quantify loss of tip metal, and for this reason ICP-MS were discontinued for the 1200 *μ*A case.

The 1200 *μ*A stimulation far exceeds the 100 *μ*A to be used in the clinic study, and is justified by the much higher impedance and lower charge injection capacity for Utah array electrodes in vivo and desire to explore the limits of stimulation stability. Thorough electrochemical characterization of Utah arrays in rat cortex up to 25 weeks by Nguyen et al. (2026) found impedances increased by >10× and CIC decreased by 5 to 10× from 2 down to 0.2 to 0.4 mC/cm^2^; therefore, higher stimulation voltages than needed for the 100 *μ*A stimulation will potentially be required to deliver this current during the clinical study. Only one stimulated electrode (Device 2 K8) demonstrated the abrupt increase in voltage, the hallmark of electrode failure. The voltage matrices collected after this failure show little voltage on surround electrodes when K8 is stimulated, strongly suggesting current is not reaching the electrode, likely due to a broken lead wire or bond pad. All other channels with high voltages maintained the voltages across the entire paradigm, and were either unstimulated control or discarded electrodes. This is consistent with the electrodes having failed prior to starting the study, and therefore do not suggest stimulation induced damage.

The BSEM micrographs provide the most precise physical characterization of IrOx and electrode damage and used for this analysis. Three electrodes stimulated with the 1200 *μ*A paradigm from Devices 4 and 5 exhibited lost IrOx, rounded morphologies for exposed Si at the tip, and a separation between the Si and tip metal, consistent with prior reports of electrode damage Bjånes et al. (2025) Stump et al. (2025). Two damaged electrodes were from Device 4, which had the previously described ubiquitous chipped tips. The damaged electrodes had chipped tips as-received, and experienced a delaminated area of IrOx where the flake was still present in the micrograph and corrosion of the silicon shank after stimulation. Device 5 developed damage to the IrOx on the electrode tip where IrOx had delaminate from the tip prior to the study. These results indicate that damage mechanisms are active during this high-dose paradigm, but the slow rate of degradation at these extreme conditions suggest AN-USA electrodes will likely tolerate the stimulation in our 1 year clinical study.

The overall evaluation is that the devices tolerated accelerated aging and both the physiological and high-dose stimulation conditions well. Electrode impedances *activated* to low values, and with one exception stayed consistent or decreased, despite the presence of *buckled* metal and chipped tips particularly for Devices 1 - 4. Impedances in vivo will be higher, while CIC will be lower, but the data suggests there is a good chance this can be accommodated. The in vivo environment is also more complex than saline, where outcomes from in vivo are often worse than predicted by bench studies, which makes the point that better accelerated aging tests for microelectrode devices remain a critical need.

## 5 Conclusions and Future Work

The objective for the ANI program is to evaluate hearing outcomes achieved by more direct and selective stimulation of the auditory nerve with a new hearing prosthesis using a penetrating microelectrode array through a first-in-human study. Demonstrating the stability and integrity of electrodes for the ANI device is needed to enable the clinical investigation; therefore, we performed TAA and Stim-Stab testing to evaluate stability for saline soaking and electrical stimulation, respectively. Baseline physical and electrical characterization determined that the electrodes had an average surface area of 3.68 × 10^−5^ cm^2^, a typical value of Utah array, with one device noted for having a large variation. Measured electrochemical characteristics also aligned with expectation, with a 1 kHz impedance of 4.86 ± 1.81 kΩ, CSC_c_ of 25.83 ± 3.62 mC/cm^2^, and CIC of 1.70 ± 0.25 mC/cm^2^. Bench voltage transients collected using 1200 *μ*A pulses exceeded the water window, suggesting that the Stim-Stab paradigm at these conditions also were above the water window, but electrode *activation* processes made it difficult to confirm this for the study.

The primary metric for the 1.5 year equivalent TAA test was impedances of <70 kΩ, which all electrodes met at the study endpoint. The impedance of three electrodes did notably increase transiently exceeding the failure criteria before returning to passing values. OM and BSEM were used to image all electrodes before, during, and after soak testing, with OM providing useful quantification of electrode quality, and BSEM providing highly precise data. The TAA devices had statistically more initial damaged compared most Stim-Stab devices, with a buckled and poorly adhered IrOx and chipped tips being the most common damage types. Two devices showed little to no change in electrode damage, while a third device that had poor initial IrOx adhesion before the test did show delamination for 6/15 electrodes. Buckled IrOx has since been addressed by a small integration process change, which when combined with the stable electrode impedances indicates ANI device electrodes have the soak stability needed for our clinical study.

Stim-Stab testing evaluated electrode stability during 6.3 Gpulses using 100 *μ*A, the maximum we will use in vivo to match the maximum estimated charge subjects will receive during 1 year of stimulation of 252 C. We also utilized the same protocol, but with 1200 *μ*A pulses as a high-intensity paradigm, delivering 3024 C. No electrodes from the 100 *μ*A study, and one electrode from the 1200 *μ*A study had the abrupt voltage >50% voltage increase for a failed electrode, but more careful analysis of the voltage matrix suggests a broken lead wire was the cause. Given the 12× larger current amplitude of the 1200 *μ*A protocol and associated high stimulation voltages required to drive the current, their stability suggests the electrode stimulation lifetime is compatible with our clinical use. All electrodes for the 100 *μ*A case, with the exception 11 damaged by handling during imaging procedures and one with a variation in imaging perspective, where not effected by the Stim-Stab protocol, which represents the maximum current used in vivo. The 1200 *μ*A Stim-Stab resulted in 3/20 stimulated electrodes being damaged, with two of those having initial damage that likely contributed to stimulation induced damage. The data suggests these very high currents were tolerated well, and corrosion of silicon, particular the material exposed before stimulation, resulted in delamination of the IrOx tip. Outcomes from the Stim-Stab studies support that ANI electrodes have sufficient stability to be compatible with the 1 year of stimulation for our clinical study.

A number of electrodes especially from Devices 1-4 had electrode damage as received, and this damage contributed to those electrode failures observed. The initial damage has been significantly decreased by a small change in the integration process, and the results suggest further optimization of the tip metallization stability is possible. This is further supported by noting that tip metal and electrode damage that is worse compared to many bench studies was observed for cortical explants after 2.5 to 5.5 years in-dwellingBjånes et al. (2025). These outcomes suggest that improving the electrochemical characteristics and adhesion of the IrOx tip metal to the silicon electrode shanks could contribute to longer electrode lifetimes for applications such as sensory neuroprosthetics and brain-computer interfaces.

## Supplementary Material

This is a list of supplementary files associated with this preprint. Click to download.

- ANISupplemental.pdf

The online version contains supplementary material available at.

## Figures and Tables

**Fig. 1:**
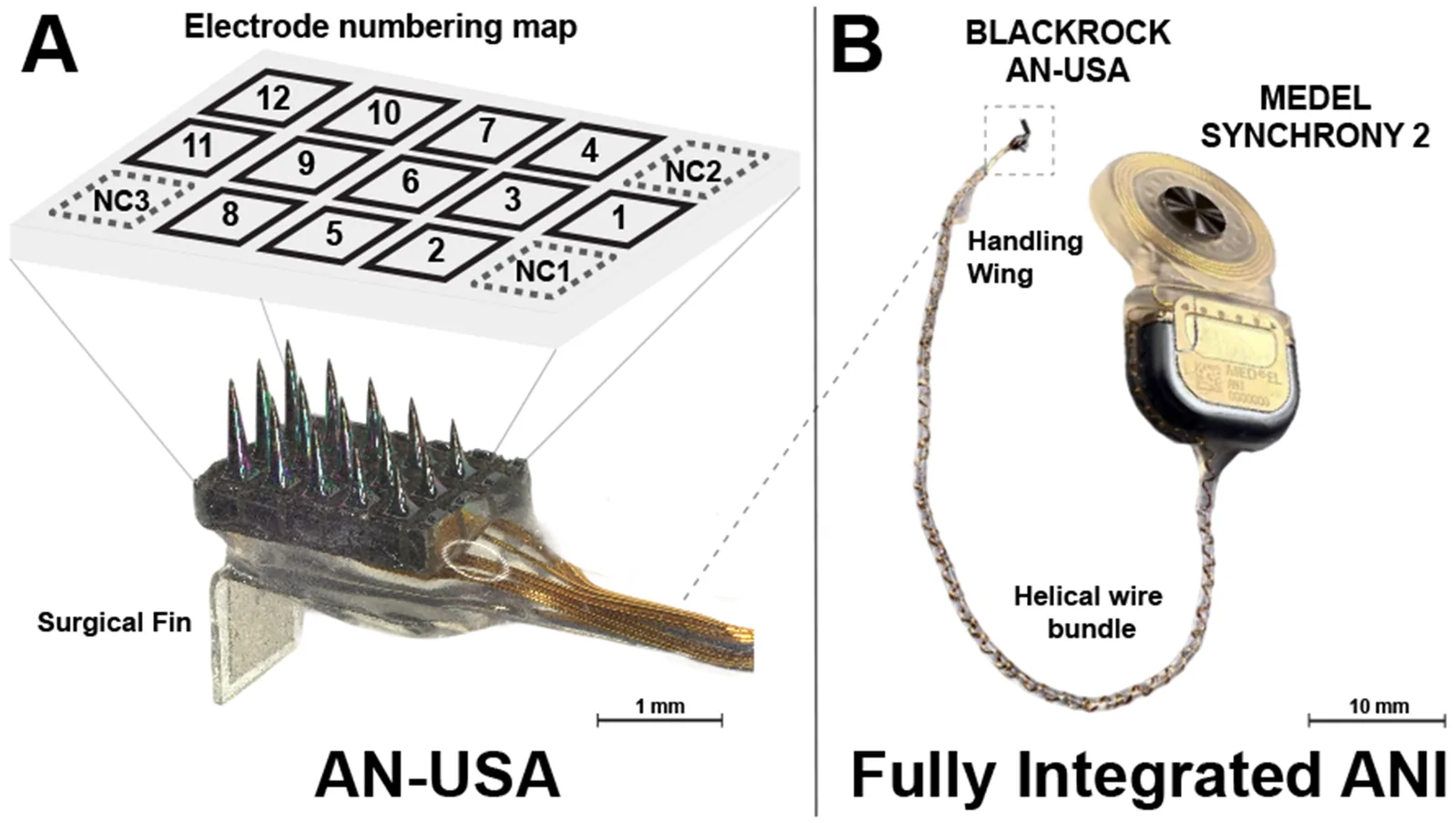
Overview images of the ANI device including an **(A)** optical micrograph of the AN-USA electrode array and a projection of the corresponding electrode mapping. Electrically connected channels are labeled 1-12, while not connected ones are labeled NC1-3. The overmolded Pt-Ir *Fin* on the array’s backside used for surgical handling is identified. **B)** Picture of a fully integrated ANI device comprised of a MEDEL SYCHRONY2 stimulator connected by the helically wound lead to the Blackrock AN-USA. A silicone *Handling Wing* located 10 mm proximal to the array is labeled.

**Fig. 2:**
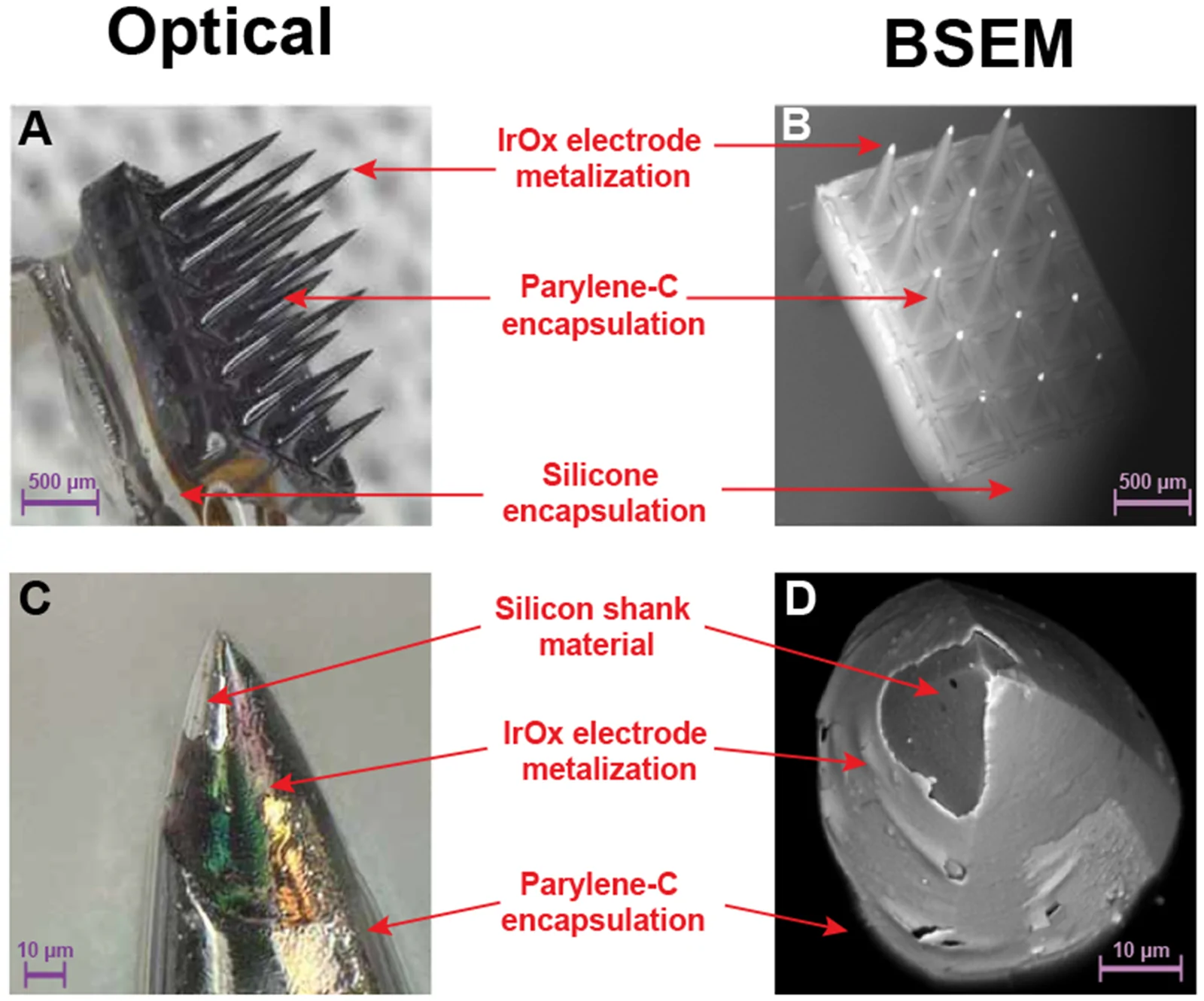
Representative optical and backscattered scanning electron microscopy (BSEM) micrographs, presenting **(A)** an overview optical and **(B)** overview BSEM micrographs of ANUSA showing all 15 electrodes. **C)** Higher magnification optical (500×) and **(D)** BSEM (1250×) micrographs of electrode tip. Red call-outs identify locations and contrast mechanisms associated with IrOx, Parylene-C, silicone, and silicon.

**Fig. 3:**
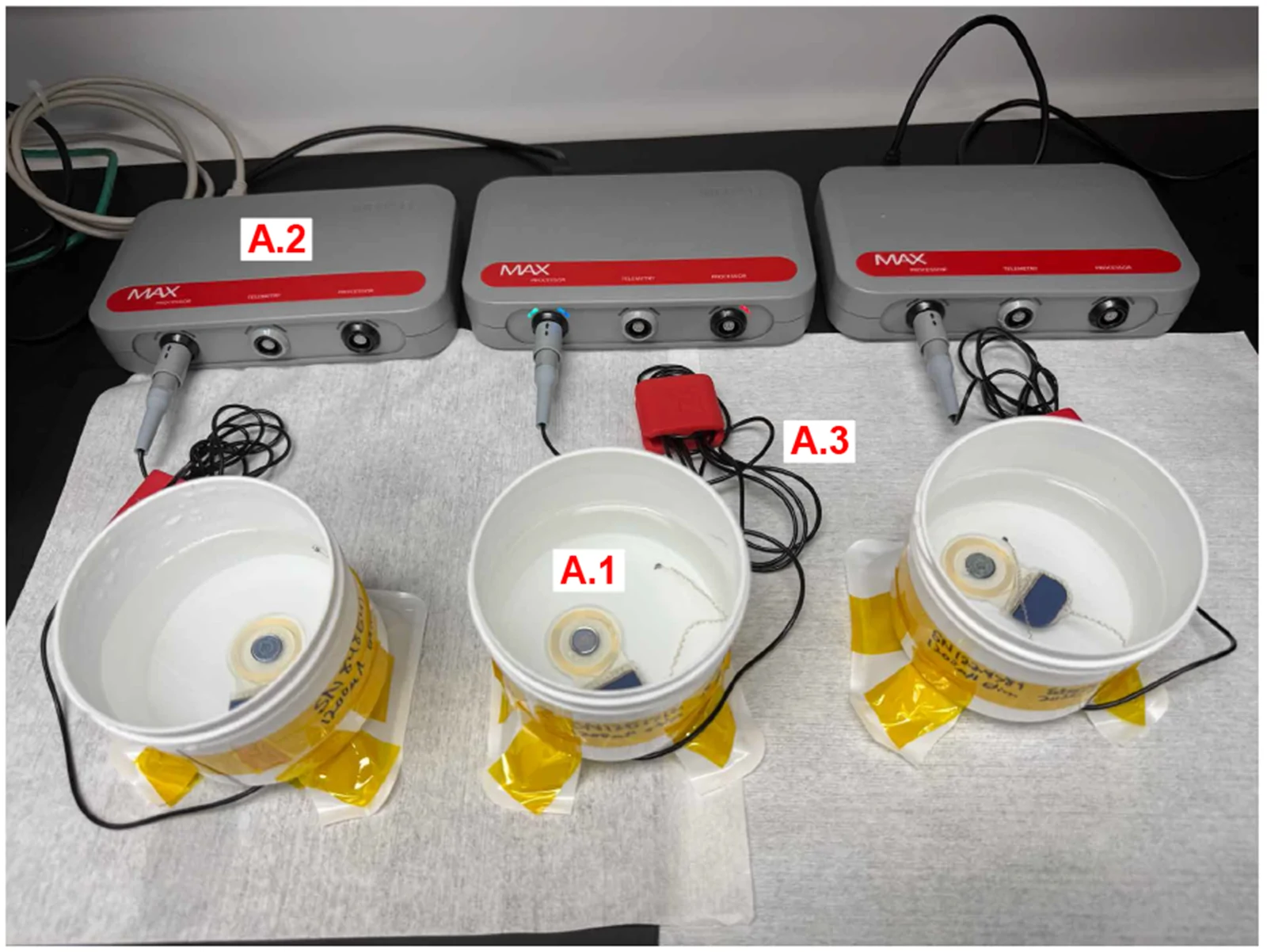
Image of three **(A.1)** ANI devices connected to **(A.2)** MAXbox interfaces for controllers using **(A.3)** magnetically attached MAXCoil-S for stimulation stability testing or TAA voltage matrix measurements. The 300 mL polypropylene containers are shown with sealing lids removed to reveal the ANI devices immersed in 150 mL 1× PBS.

**Fig. 4:**
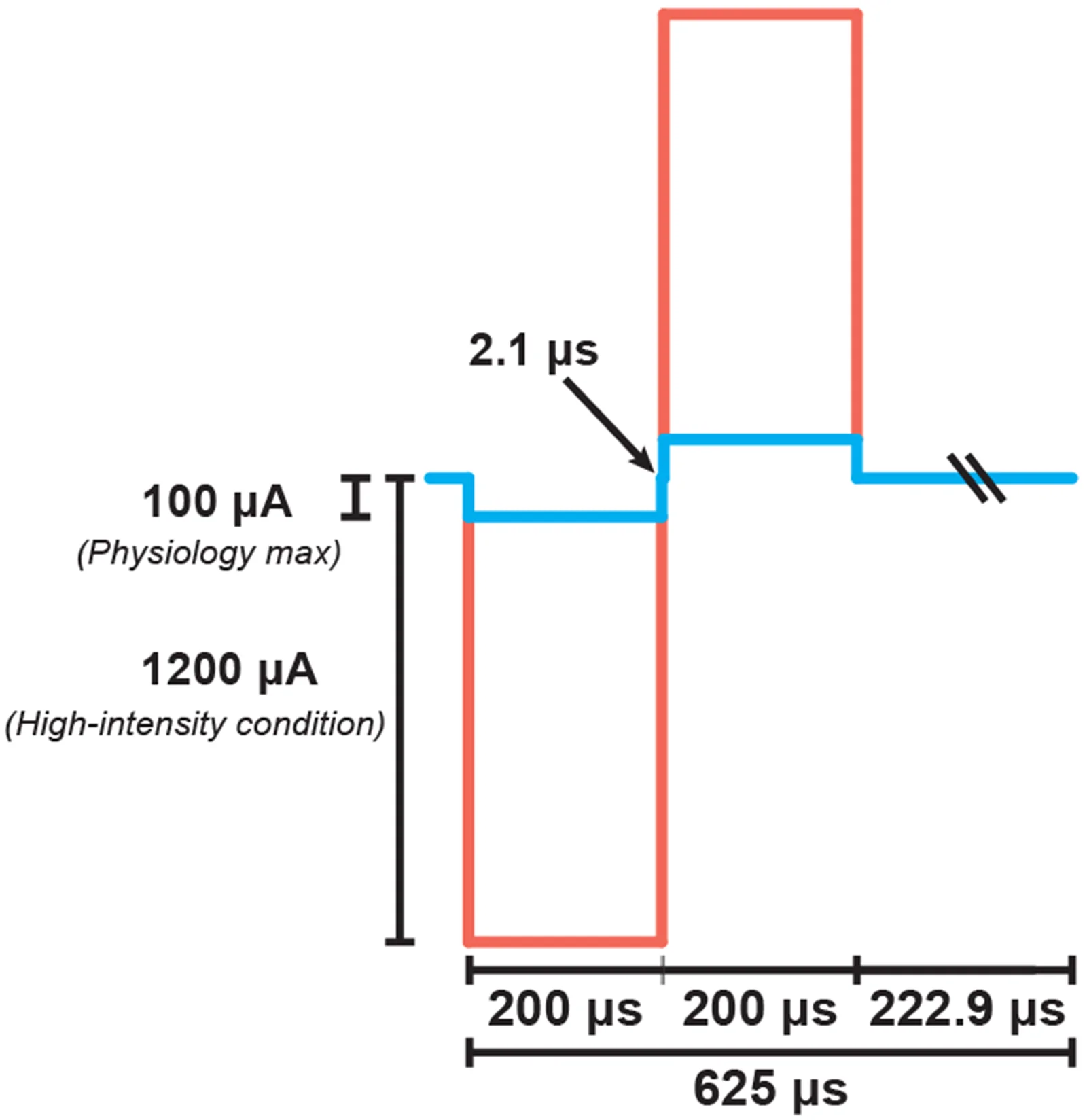
Accurately scaled stimulation waveforms used for the 100 and 1200 *μ*A stimulation paradigms. Waveform parameters consisted of biphasic charge-balanced cathodic leading pulses at 1600 pps with a 200 *μ*s phase duration and 2.1 *μ*s interphase gap.

**Fig. 5:**
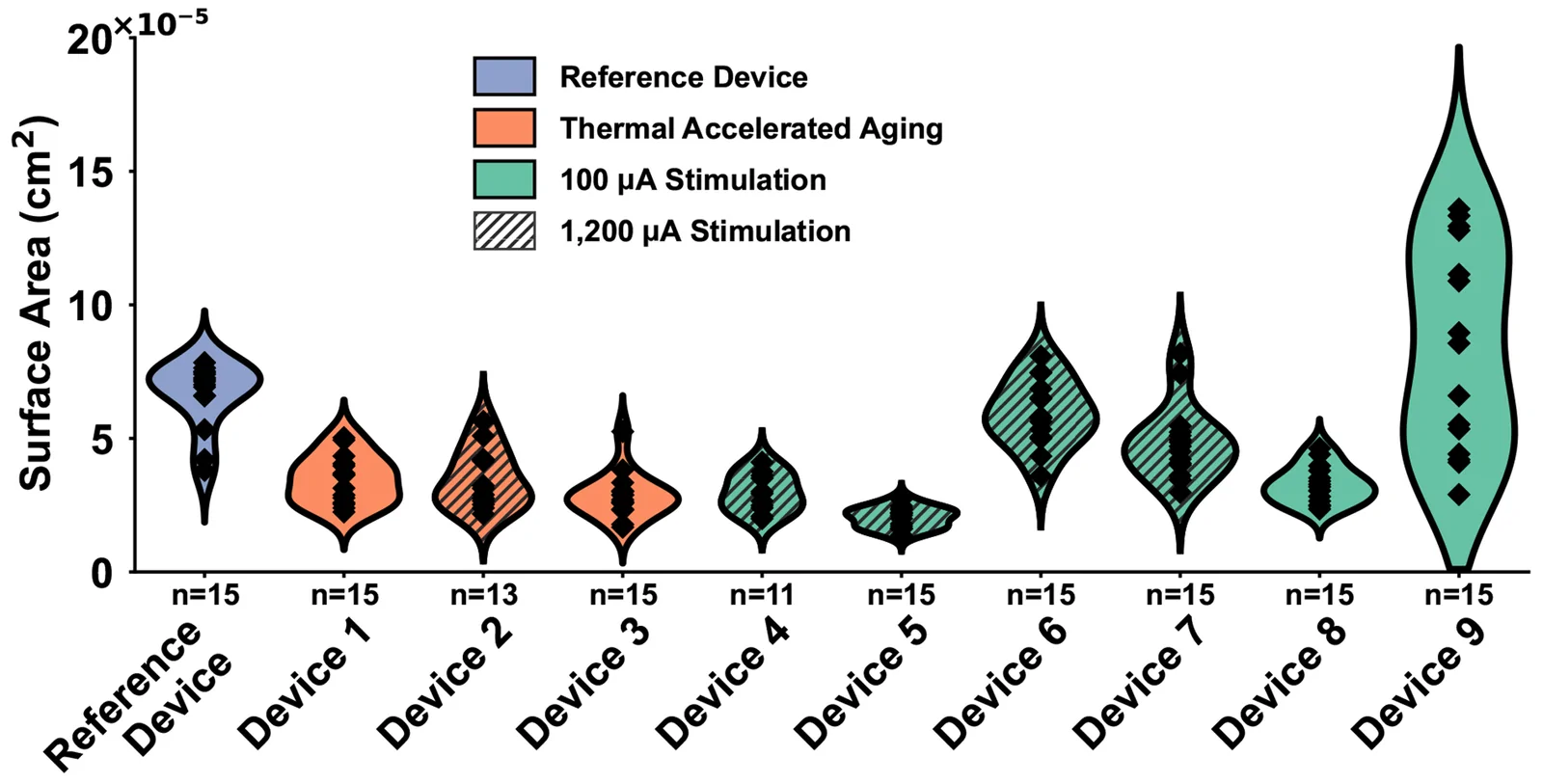
Electrode tip geometrical surface area for all nine devices and baseline AN-USA assembly are presented as a violin plot. The average geometric surface area was 3.68 × 10^−5^ cm^2^. Color indicates the test protocol each devices was used for, and hatching indicates the five devices that underwent the additional 1200 *μ*A stimulation paradigm.

**Fig. 6:**
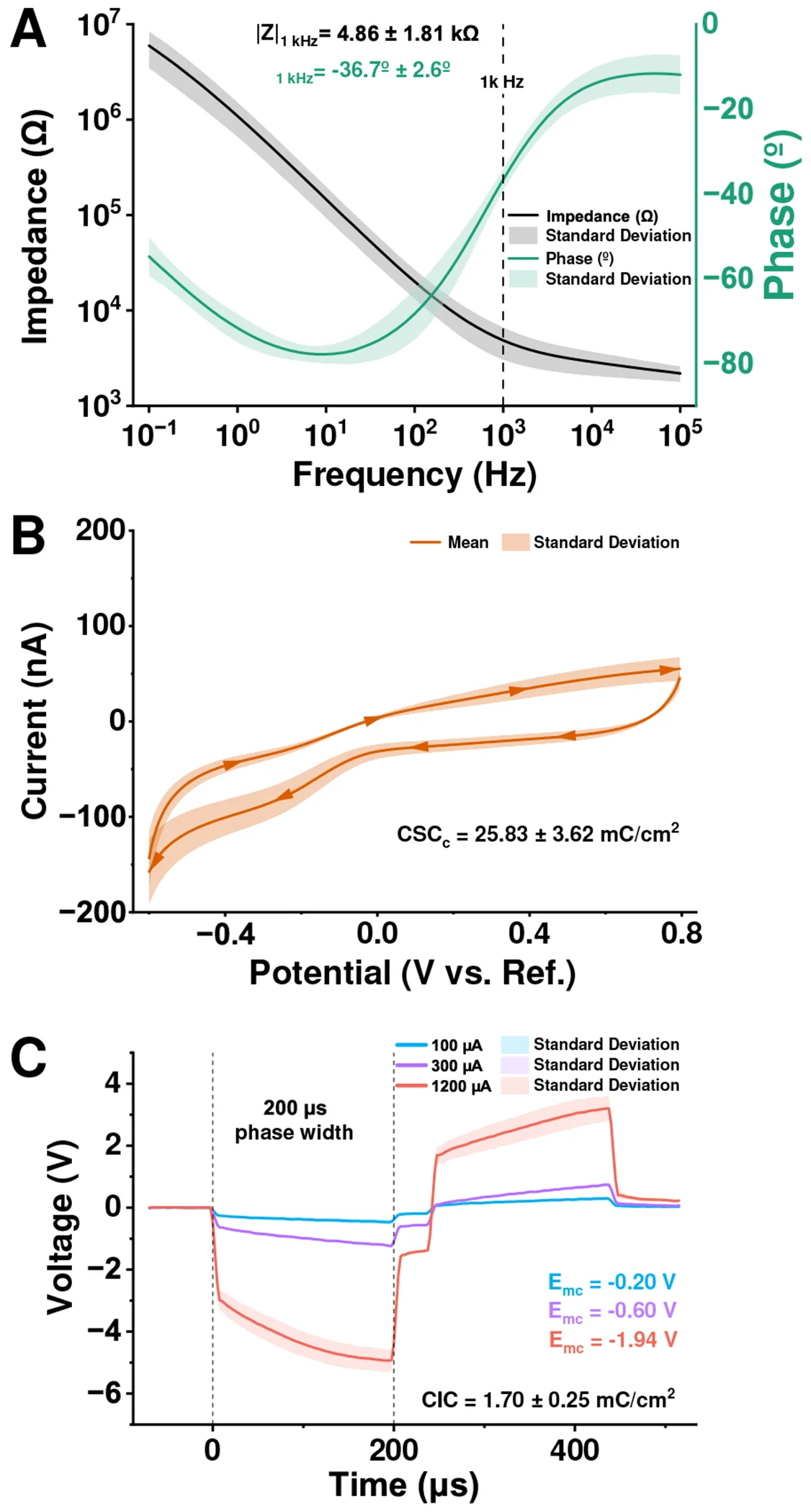
Electrochemical characteristics of reference ANI assembly electrodes (n=8). **A)** Electrochemical impedance spectroscopy (EIS) as a combined Bode plotted with average magnitude and phase a a function of frequency, with a 1 kHz the impedance of 4.86 ± 1.81 kΩ and phase of–36.7 ± 2.6°. **B)** Averaged cyclic voltammetry (CV) waveform of potential sweeps within water window −0.6 to +0.8 V vs Ag—AgCl, used to quantify a CSC_c_ of 25.83 ± 3.62 mC/cm^2^ . **C)** Average voltage transient (VT) for the 100 *μ*A (blue) and 1200 *μ*A (red) stimulation conditions. A 300 *μ*A (purple) waveform is shown where *E_mc_* reached the water window (−0.6 V vs Ag—AgCl) used to quantify an average CIC of 1.70 ± 0.25 mC/cm^2^.

**Fig. 7:**
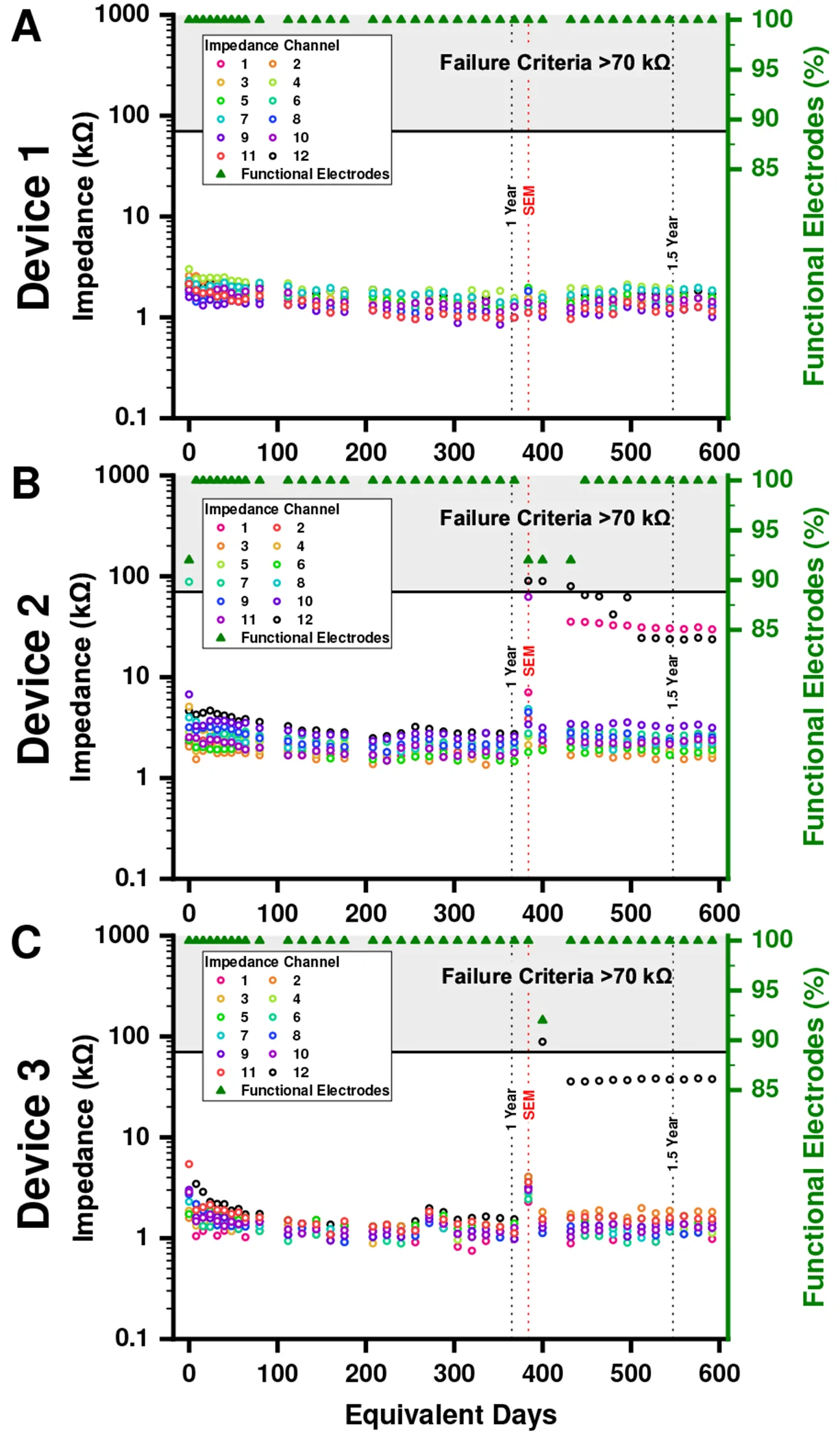
Thermal Accelerated Aging (TAA) longitudinal impedances plotted for an equivalent 1.5 years, where all electrodes remained below 70 kΩ failure criteria at the endpoint. **A)** Device 1 electrodes had steady impedances with an average 1.54 ± 0.20 kΩ, similar for all devices, while for **B)** Device 2, electrodes 7 and 12 transiently exceeded 70 kΩ failure criteria before returning to high but passing values, similar to **C)** Device 3 electrode 12.

**Fig. 8:**
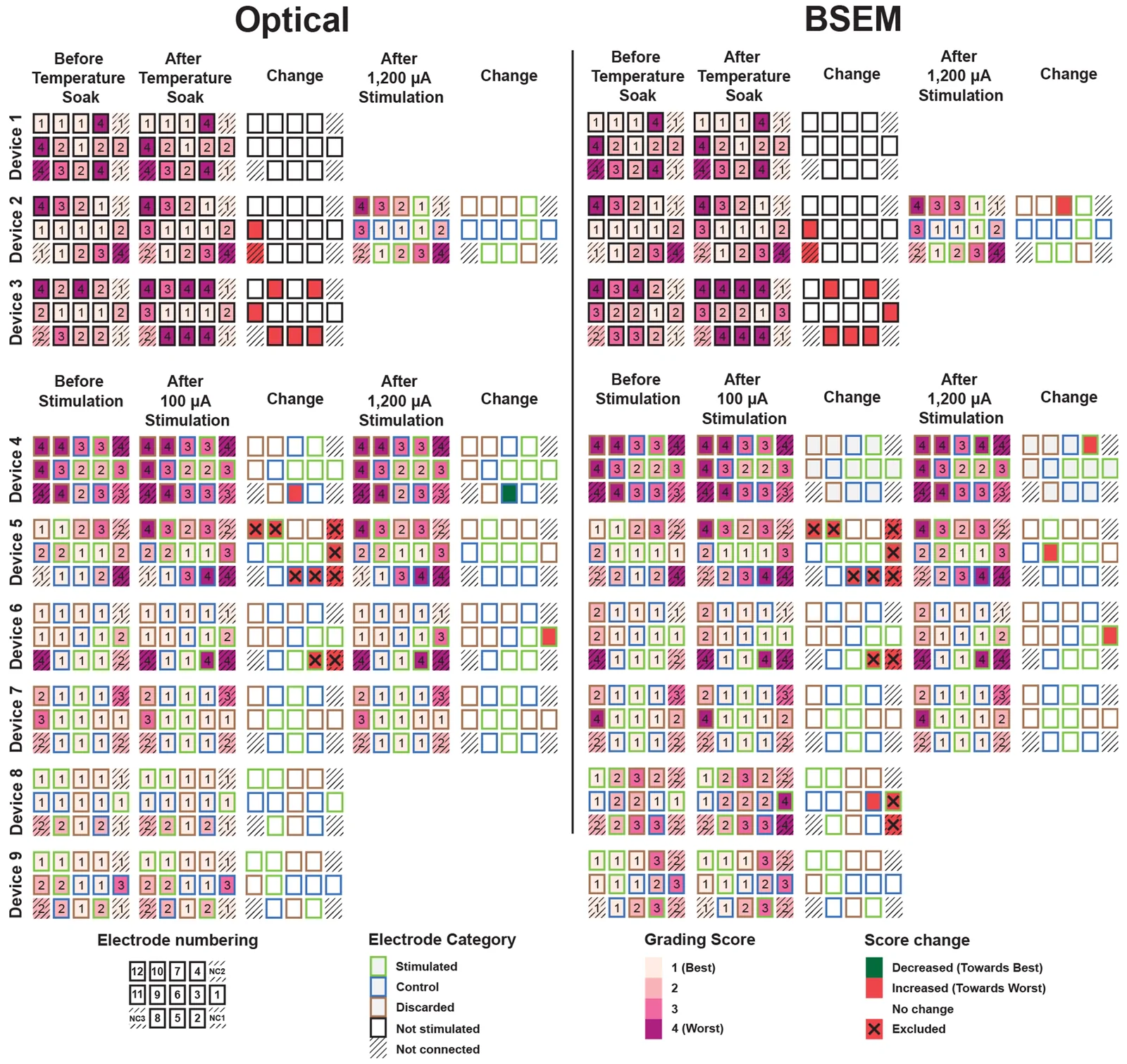
Heatmaps of scores from 1 = Best (light pink) to 4 = Worst (dark pink) based on (left) optical and (right) electron imaging from before and after all testing paradigms. Electrode numbering for stimulated paradigm devices follows the same convention as Figure 1a and is schematically provided. Boarder colors represent stimulated electrodes (green), control (blue), discarded (brown), not stimulated (black), and not connected (hatched). Heatmaps quantifying the score *Change* during a paradigm use green squares to indicate a decreased score, red for an increase, and red with an *X* for an excluded electrode due to handling damage. All electrode micrographs can be found in supplemental materials S5-S13.

**Fig. 9:**
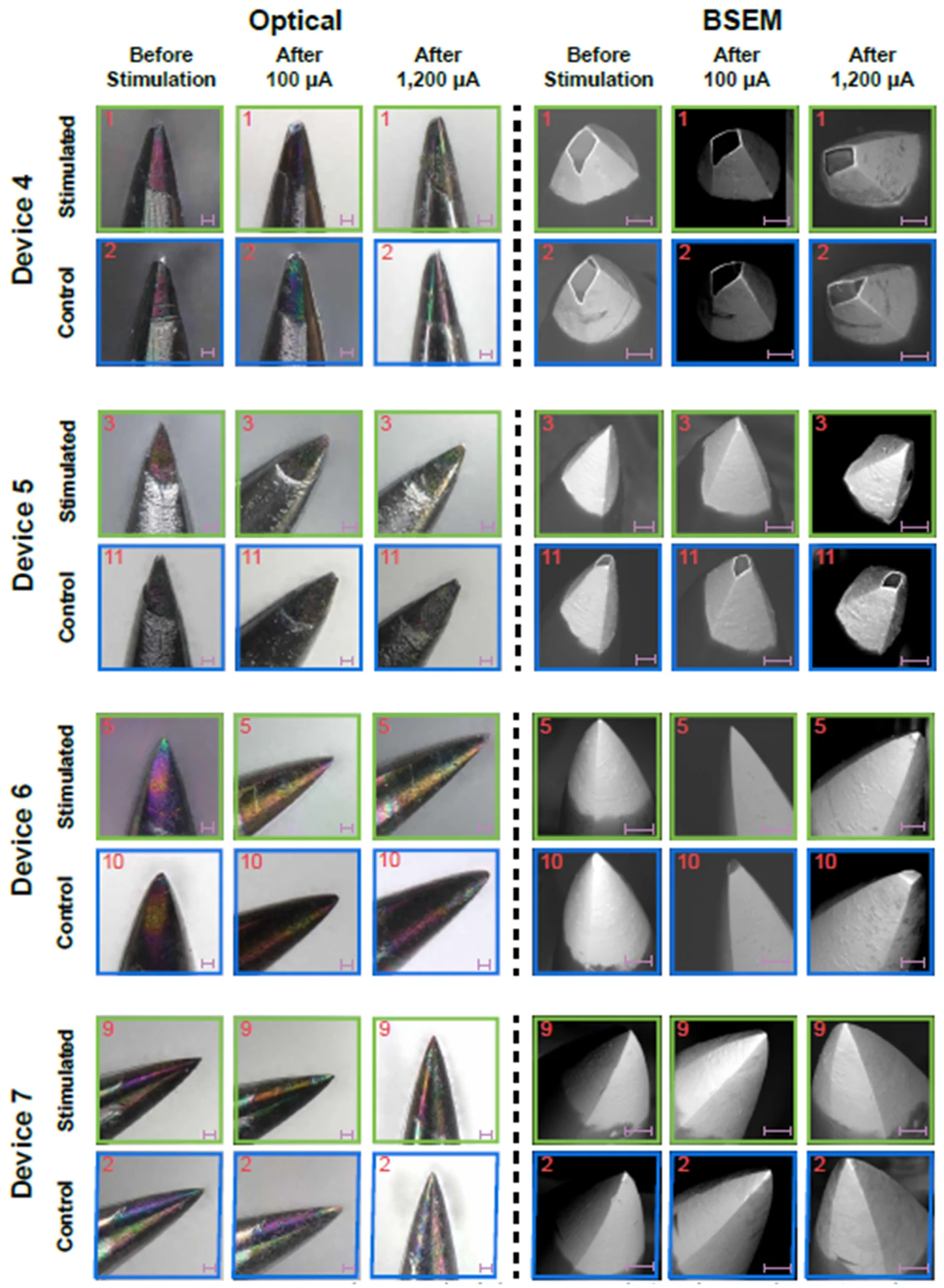
Representative before and after stimulation optical and electron micrographs from the 100 and 1200 *μ*A paradigms, including stimulated and control electrodes, where all scale bars are 10 *μ*m. The imaging indicates that neither the paradigm’s stimulation intensity was sufficient to produce a measurable impact on electrode metallization, which largely remained unaltered. The stimulated electrod from device 4 presents as a chipped electrode tips before stimulation where a degree of degradation occurs during the 1200 *μ*A paradigm resulting in corrosion of the exposed silicon and a small degree of IrOx delamination.

**Fig. 10:**
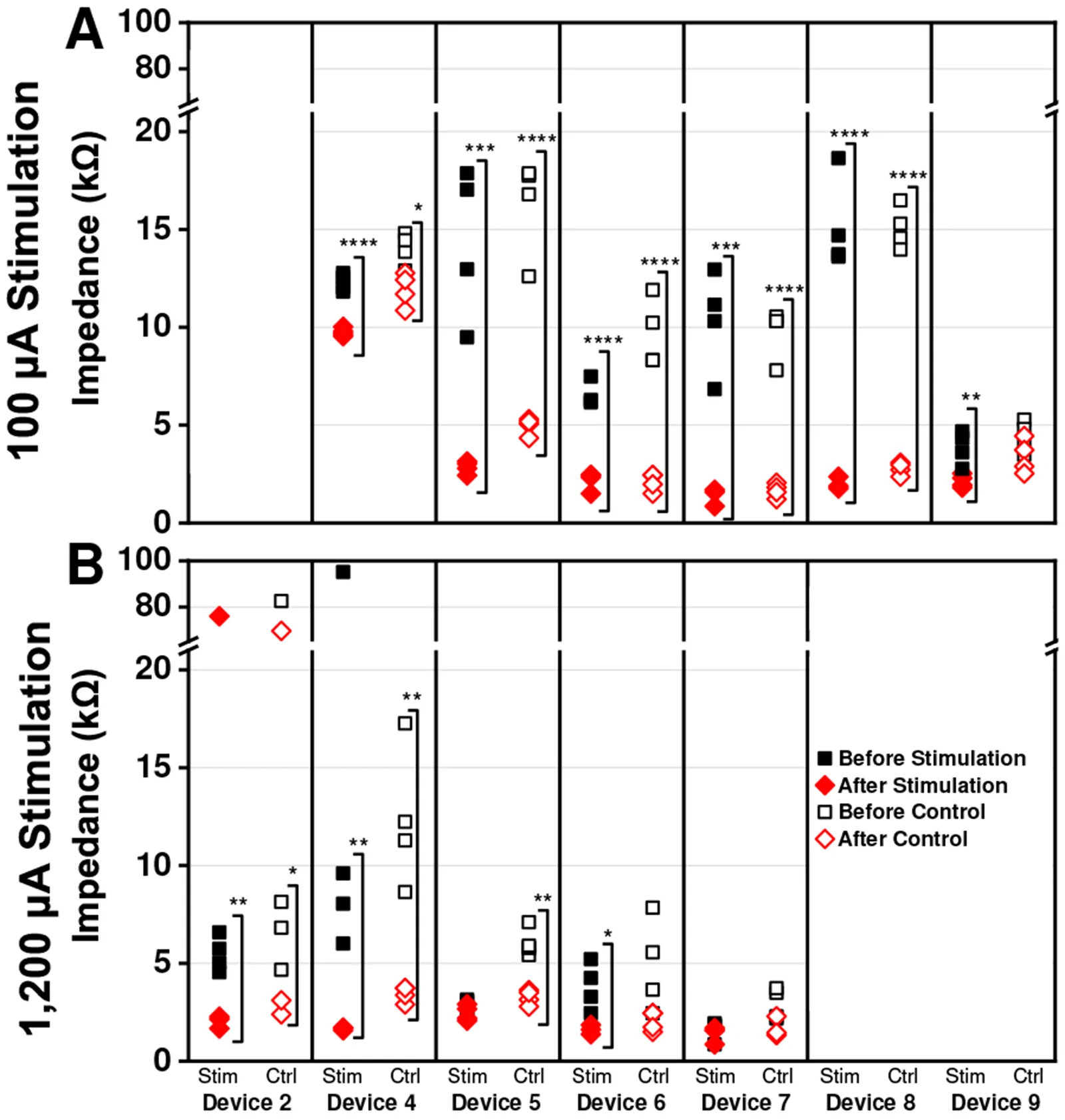
Before and after impedances from the **(A)** 100 and **(B)** 1200 *μ*A stimulation paradigm. In both stimulation conditions, all electrode impedances were initially higher with more variation, which typically decreased to lower to 2-3 kΩ values with less variation after stimulation. Statistically significant changes from before to after stimulation are identified using standard notation for significance. Stimulated and control categories each had n=4 electrodes.

**Fig. 11:**
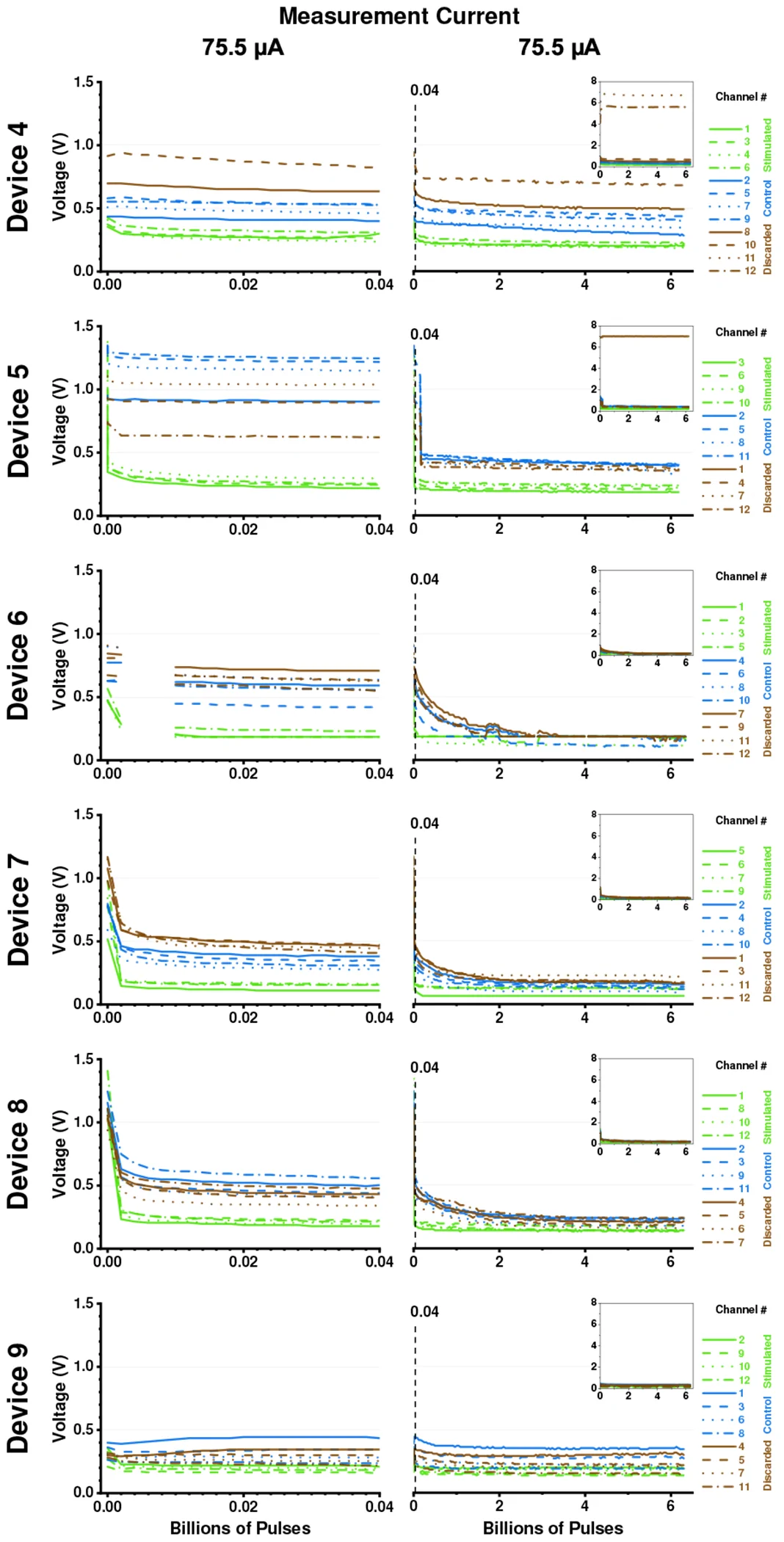
Longitudinal stimulation voltage data up to 1.5 V for the 100 *μ*A paradigm across (left) the initial 40 Mpulses and (right) the full 6.3 Gpulses for each device. Measurements are collected with a 75.5 *μ*A current to conform with measurements from Maestro. The inset plot presents voltage data up to 8 V to capture open-circuit or high impedance channels. The traces for channels are categorized by stimulated (green), control (blue), and discarded (brown) for the 12 connected electrodes.

**Fig. 12:**
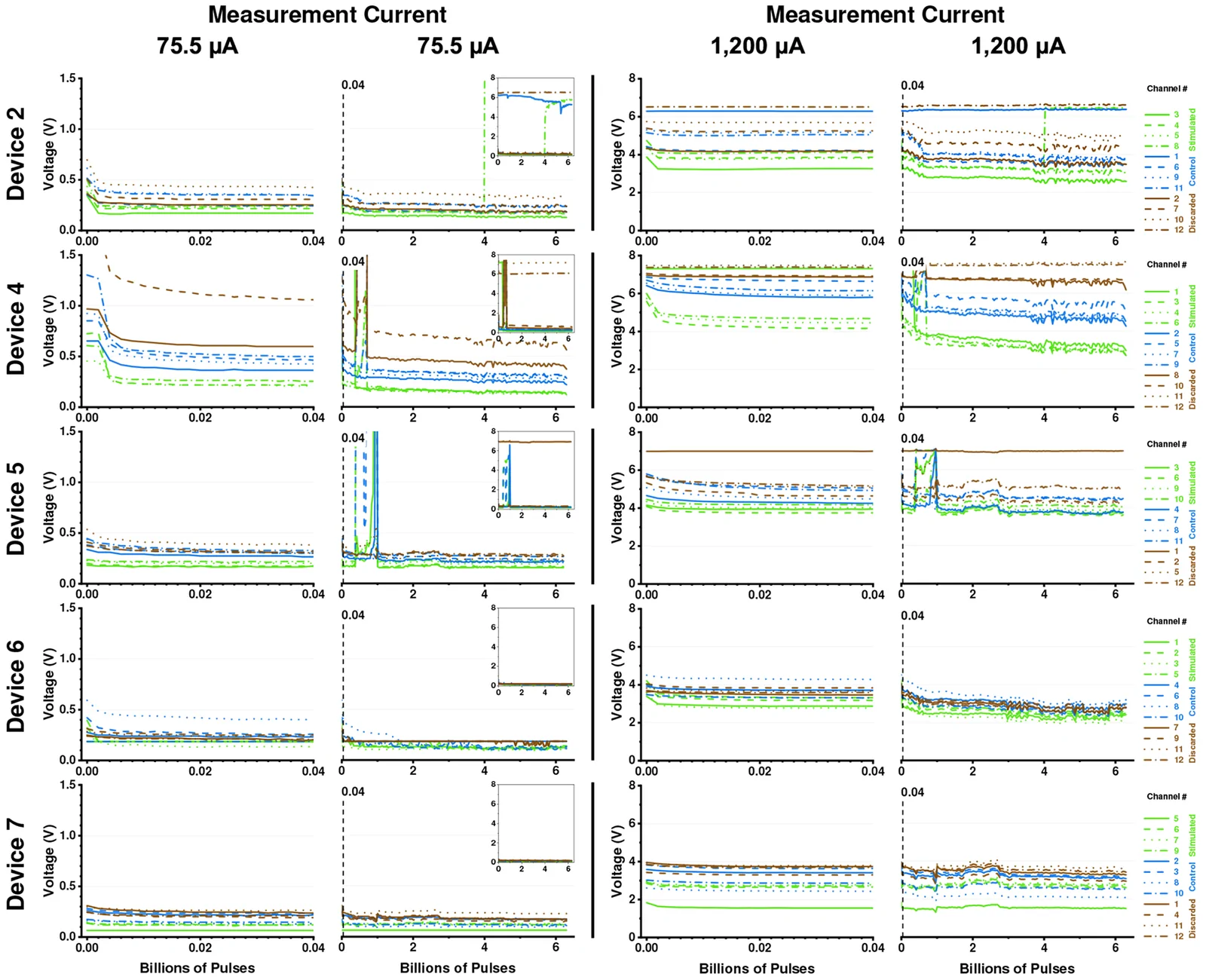
Longitudinal stimulation voltages for the 1200 *μ*A Stim-Stab paradigm with (left two columns) measurements collected using 75.5 *μ*A pulses and (right two columns) 1200 *μ*A pulses for each device. The plots measured with 75.5 *μ*A facilitate observing abrupt voltage increases, while measurements using the actual 1200 *μ*A stimulation intensity highlight the substantial driving voltages required during the paradigm for some devices. Each measurement condition include plots across 40 Mpulses and 6.3 Gpulses, and the 75.5 *μ*A plot includes and inset that presents voltage data up to 8 V to capture open-circuit or high impedance channels. The traces for channels are categorized by stimulated (green), control (blue), and discarded (brown) for the 12 connected electrodes.

**Table 1:** List of all AN-USA devices and their respective number of electrodes (n) used in temperature accelerated aging and/or stimulation-stability testing.

				Stim-Stab	
	Device #	Serial #	TAA (n)	100 *μ*A (n)	1200 *μ*A (n)
**TAA** 67°C/77°C 1.5 yr equiv.	Device 1	1226241	12		
	Device 2	1259789	12		4
	Device 3	1256329	12		

**Stim-Stab** 6.3B pulses 1 yr equiv.	Device 4	848541		4	4
	Device 5	1251558		4	4
	Device 6	1251712		4	4
	Device 7	1253504		4	4
	Device 8	1228693		4	
	Device 9	1229807		4	

**Table 2:** ICP-MS operating conditions

Plasma Frequency	27.14 MHz
RF Power	1550 W
RF Matching	1.50 V
Sample Depth	8.0 mm
Plasma gas flow rate	15.0 L/min
Auxiliary gas flow rate	0.90 L/min
Nebulizer gas flow rate	1.07 L/min
Make-up gas flow	0.00 L/min
Nebulizer pump	0.10 rps
Spray chamber	Scott type
Cones	Nickel
Sensitivity	9.5 kcps for Li 1 *μ*g/L 29.8 kcps for Y 1 *μ*g/L 20.6 kcps for Tl 1 *μ*g/L

**Table 3:** ICP-MS measured concentrations (ppb) of metal ions in PBS soak samples. All stimulated AN-USA (n=6) had Pt detected above LoQ, and Device 4 and 6 had Ti detected above LoQ. All other elemental traces from Ir and Au were not able to be quantified.

	^47^Ti	^191^Ir	^195^Pt	^197^Au
LoD	0.51	0.049	0.04	1.5
LoQ	1.5	0.15	0.12	4.4

**Samples in polypropylene vials (n=6)**				
Device 4	4.5	0.015	0.90	0.20
Device 5	0.84	0.011	1.3	0.13
Device 6	4.2	0.019	2.2	0.20
Device 7	0.027	0.0080	0.89	0.15
Device 8	0.49	0.014	0.68	1.0
Device 9	0.44	0.0068	0.44	0.35

## Data Availability

All data is available upon reasonable request.

## Abbreviations

AN: Auditory Nerve
ANI: Auditory Nerve Interface
UA: Utah Array
USA: Utah Slant Array
AN-USA: Auditory Nerve - Utah Slant Array
PBS: Phosphate-Buffered Saline
CI: Cochlear Implant
TAA: Thermal Accelerated Aging
Stim-Stab: Stimulation Stability
VMM: Voltage Matrix Measurement
OM: Optical Microscopy
SEM: Scanning Electron Microscopy
BSEM: Backscattered Scanning Electron Microscopy
MAXBox: MAX Programming Interface
ICP-MS: Inductively Coupled Plasma – Mass Spectrometry
LoD: Level of Detection
LoQ: Level of Quantification
SIROF: Sputtered Iridium Oxide Film
Mpulses: 1 × 10^6^ pulses
Gpulses: 1 × 10^9^ pulses
EIS: Electrochemical Impedance Spectroscopy
CV: Cyclic Voltammetry
VT: Voltage Transient
GSA: Geometrical Surface Area
